# Narrative Review of Digital Twins in the Health Domain: Development, Application, and Evidence Consolidation

**DOI:** 10.3390/medsci14020330

**Published:** 2026-06-18

**Authors:** Daniele Giansanti, Claudia Cosenza

**Affiliations:** 1 Centro Nazionale Intelligenza Artificiale e Tecnologie Innovative per la Salute, Istituto Superiore di Sanità, Via Regina Elena 299, 00161 Rome, Italy; 2 Facoltà di Medicina e Psicologia, Università Sapienza, Ospedale S. Andrea, Via di Grottarossa 1035, 00189 Rome, Italy; claudiacosenza02@libero.it

**Keywords:** digital twin, AI, personalized medicine, precision medicine

## Abstract

**Background:** Digital twins and patient-specific computational models are emerging technologies in healthcare, enabling predictive, personalized, and adaptive interventions. Their integration with artificial intelligence (AI) facilitates the simulation of clinical scenarios, optimization of treatment strategies, and advancement of precision medicine. Despite growing interest, the evidence base is still evolving, highlighting the need for a comprehensive synthesis to identify current trends, applications, and gaps. **Methods:** A narrative review was conducted using PubMed, Web of Science, and Scopus to identify relevant literature on digital twins in healthcare. Priority was given to systematic reviews and meta-analyses in the selection process. From this process, 28 studies were selected for in-depth analysis, and their findings were complemented by primary research and conceptual, and synthesized evidence to capture emerging trends and real-world applications. **Results and Discussion:** The analysis revealed that digital twins are increasingly applied for patient-specific monitoring, predictive simulations, and adaptive interventions. Integration with AI enhances their ability to model complex clinical scenarios and support precision medicine. While the selected systematic reviews provide consolidated evidence of established applications, the complementary analysis indicates that these studies actively contribute to stabilizing clinical evidence, consolidating knowledge, and enabling the development of more robust patient-specific strategies. **Conclusions:** Digital twins are progressively shaping patient-centered healthcare by combining AI-driven simulations with clinical insights. Current research is not only consolidating existing evidence but also exploring novel applications, underscoring the potential of digital twins to enhance precision medicine. Further studies are required to fully integrate these technologies into routine clinical practice.

## 1. Introduction

### 1.1. Digital Twins: Conceptual Foundations Across Domains and Applications in Healthcare

The concept of digital twins is characterized by a persistent lack of terminological and conceptual consensus across domains. As recently highlighted in a large-scale analysis of over 15,000 scientific publications, “Digital Twin (DT) is a widely used but still fuzzy term” with definitions that differ significantly depending on the application context, including manufacturing, built environments, and urban systems [1]. In particular, the same study shows that even within closely related fields, the core components attributed to digital twins vary substantially, and no universally accepted definition has yet been established, indicating that the concept remains in an evolving state rather than a stabilized paradigm [1].

Despite this variability, converging evidence suggests the presence of shared foundational elements across definitions, including the representation of a physical or real-world entity, continuous or periodic data updating, and the capacity to support monitoring, analysis, or decision processes. However, the relative importance of features such as real-time interaction, bidirectional data flow, and predictive simulation differs across domains and maturity levels of implementation [1].

In this context, efforts have been made to move toward a more unified and formal interpretation of the concept. In particular, Emmert-Streib et al. propose a data science-based unification of digital twins, defining them as open dynamical systems with updating mechanisms, embedded within a broader digital twin system (DTS) that enables both simulation-based data generation and decision support processes [2]. Within this framework, a digital twin can be interpreted as a complex adaptive system designed to generate and continuously refine representations of its physical counterpart through iterative data-driven updates, while the surrounding system provides analytical and decision-making functionality based on the generated data [2].

This formulation further clarifies a key conceptual distinction that remains central in the literature: the difference between a mere simulation or mathematical model and a digital twin as an adaptive, evolving system. Importantly, it also emphasizes that the value of digital twins lies not only in representation, but in their ability to continuously integrate data, generate behaviorally consistent outputs, and support higher-level reasoning and decision processes across application domains.

Within this progressively structured conceptual landscape, the application of digital twins in the health domain has emerged as one of the most rapidly evolving and multidimensional areas of research. Their increasing adoption reflects the convergence of advances in data science, artificial intelligence, and biomedical informatics, which together enable the construction of patient-specific digital representations aimed at supporting clinical understanding, prediction, and decision-making [3].

At the same time, the literature consistently highlights that healthcare digital twins are still in an early and heterogeneous phase of development. Rather than constituting a mature and unified technology, current implementations range from highly specific organ-level models to broader patient-centric predictive systems. A scoping analysis of proposed patient digital twins shows that most reported systems remain at preclinical stages, with substantial variability in their intended use, structural design, and degree of interaction with real-time patient data [4]. This variability is also reflected in the underlying data architectures, which differ in the extent to which they incorporate imaging, clinical records, laboratory results, and wearable sensor streams, as well as in how they manage unidirectional or bidirectional data flows between patient and model [4].

To address this fragmentation, several authors have proposed interpretative frameworks aimed at structuring the landscape of healthcare digital twins. These frameworks suggest that current systems can be broadly understood along a continuum ranging from simulation-oriented models, primarily used for scenario testing and predictive analysis, to monitoring-oriented systems that continuously ingest patient data, up to more exploratory constructs that remain loosely coupled with individual patients and are mainly used for research purposes [5]. This perspective highlights that the notion of a digital twin in healthcare does not correspond to a single technological artifact, but rather to a family of approaches with differing levels of clinical integration and operational maturity.

From a clinical and methodological perspective, healthcare digital twins are increasingly enabled by the integration of multimodal patient data and advanced computational techniques. Electronic health records, imaging data, laboratory values, and wearable device outputs are commonly integrated and processed through artificial intelligence and simulation-based models, allowing the generation of individualized predictions and patient-specific insights [6]. Evidence across multiple clinical domains indicates that such approaches can support improved risk stratification, enhanced treatment planning, and more adaptive disease management strategies, particularly in complex chronic and multi-factorial conditions [6].

At a broader system level, institutional initiatives such as the European Virtual Human Twins framework further reinforce the strategic relevance of this concept. In this context, virtual human twins are defined as digital representations of human health or disease states that operate across multiple levels of biological organization, from cells to tissues, organs, and organ systems. These models are intended to support clinical decision-making, optimize care pathways, and enable the simulation of interventions in controlled virtual environments, thereby extending the scope of personalized medicine toward scalable and interoperable digital infrastructures [7].

Importantly, healthcare digital twins are increasingly conceptualized not merely as predictive tools, but as integrated systems that combine monitoring, simulation, optimization, and decision-support functionalities. This system-oriented view emphasizes their role as socio-technical constructs that depend on continuous data acquisition, model updating, and computational inference, rather than static representations derived from single time-point data [8].

In operational terms, a digital twin in healthcare can therefore be understood as a dynamic, continuously updated digital representation of a patient or biological system that maintains an active coupling with real-world data streams. This coupling enables iterative refinement, scenario simulation, and predictive reasoning in near real time, distinguishing digital twins from conventional retrospective models or static data-driven approaches. Their defining value lies in their capacity to evolve alongside the patient, supporting adaptive, context-aware, and increasingly personalized clinical decision-making processes [9].

### 1.2. Digital Twins in the Health Domain: Current Applications and Methodological Challenges

Within the health domain, digital twins are increasingly recognized as a promising extension of computational modeling approaches, leveraging advances in data-driven methods, artificial intelligence, and multimodal health data integration to support more precise, adaptive, and individualized care [10,11,12,13,14,15,16]. From a methodological perspective, these developments are strongly enabled by the increasing availability of large-scale and heterogeneous health datasets, including electronic health records, imaging data, wearable sensor streams, and longitudinal clinical registries, which together allow the construction of computational representations capable of capturing complex and dynamic patient trajectories [11,16]. In this context, machine learning and deep learning approaches have demonstrated particular relevance in extracting clinically meaningful patterns from high-dimensional health data, supporting risk prediction, outcome estimation, and decision support in diverse clinical scenarios [11]. More broadly, digital twins in healthcare are conceptualized as integrative and continuously evolving systems that aim to bridge biomedical research, clinical decision-making, and healthcare system optimization, with applications spanning prevention, diagnosis, therapy personalization, and long-term disease management [13,14].

Beyond individual patient care, the digital twin paradigm is also increasingly associated with broader transformations in healthcare systems and public health strategies, reflecting its potential role in enabling learning health systems and supporting data-driven healthcare innovation [13,14]. In particular, recent perspectives highlight the relevance of digital twins as global models for preventive and personalized medicine, where continuous integration of clinical and biological data can support adaptive decision-making across different levels of healthcare delivery [13]. Similarly, the extension of digital twin applications toward population health and well-being further emphasizes their potential to operate across multiple scales, from individual physiology to healthcare system dynamics, thereby reinforcing their positioning as multi-layered computational infrastructures rather than isolated predictive tools [14,17,18]. This expansion of scope also aligns with emerging views that interpret digital twins not only as patient-specific models but as enabling technologies for precision public health and health system optimization [14].

Despite this rapid conceptual and technological expansion, the current state of healthcare digital twins remains highly heterogeneous and methodologically fragmented. Evidence from systematic and scoping reviews consistently indicates that most existing implementations are still at early or preclinical stages, with limited clinical translation and substantial variability in definitions, modeling strategies, and validation practices [3,4]. In particular, reported systems differ widely in their level of granularity, ranging from highly specific organ-level models (such as cardiovascular or neurological simulations) to broader patient-centric constructs and exploratory research frameworks that are not always directly coupled with individual patients [4,5]. This diversity is further reflected in differences in data integration strategies, which may include combinations of clinical records, imaging, laboratory values, and wearable device data, as well as in the degree of temporal updating and feedback between patient and digital representation [4,19,20]. As a consequence, digital twins in healthcare cannot yet be considered a unified technological class, but rather a spectrum of approaches characterized by differing assumptions, computational foundations, and intended clinical roles [5,19].

To address this heterogeneity, recent literature has proposed structured classification systems and conceptual frameworks aimed at organizing the rapidly expanding landscape of healthcare digital twins. These approaches suggest that current implementations can be systematically categorized according to multiple dimensions, including hierarchical level (e.g., cellular, organ, patient, population), functional purpose (e.g., simulation, monitoring, prediction, optimization), and maturity stage (from conceptual prototypes to partially validated clinical tools) [5,8]. Such taxonomies help to clarify the conceptual dispersion observed in the field and provide a more coherent basis for comparing heterogeneous models and applications. Complementary conceptual perspectives further emphasize that digital twins should be understood as socio-technical systems rather than purely computational artifacts, as their functionality depends on continuous data acquisition, model updating, and integration within healthcare workflows [20]. In parallel, their relevance is increasingly extended beyond clinical medicine to include health and well-being applications, reinforcing their potential role in preventive strategies, personalized care pathways, and broader health system optimization [18]. Nevertheless, despite these advances in conceptual structuring, significant challenges remain related to model validation, interoperability, data standardization, ethical governance, and real-world implementation, which continue to limit their large-scale adoption in clinical practice [17,19].

### 1.3. Scope, Aim, and Rationale of the Narrative Review

The rapidly expanding literature on digital twins in the health domain is characterized by substantial conceptual heterogeneity, rapidly evolving technological frameworks, and variability in methodological approaches, clinical applications, and levels of implementation maturity. As discussed in the previous sections, the field spans a broad continuum of models, ranging from simulation-based computational approaches to continuously updated patient-specific systems integrating multimodal biomedical data. At the same time, definitions, architectures, and intended clinical uses remain fragmented across studies and disciplines, reflecting the early and still consolidating nature of this domain.

Given this level of heterogeneity, a purely systematic review design based on narrowly defined eligibility criteria would not adequately capture the breadth and conceptual evolution of the field. In particular, strict inclusion frameworks may risk excluding relevant conceptual contributions, methodological developments, and cross-domain perspectives that are essential for understanding the current state and trajectory of digital twin research in healthcare.

For this reason, a narrative review approach was adopted, allowing a synthesis of a heterogeneous and rapidly evolving body of literature.

To maintain methodological rigor, the review prioritizes secondary high-level evidence, with greater emphasis on systematic reviews and meta-analyses, complemented by selected reviews and foundational studies when necessary to clarify emerging or conceptually relevant developments.

The aim of this narrative review is to critically examine the current landscape of digital twins in the health domain, focusing on conceptual definitions, enabling computational and data-driven approaches, clinical and well-being applications, methodological challenges, and key limitations related to data integration, interpretability, and translational validity in medical settings.

## 2. Design of the Study

This narrative review focuses on the application and conceptual development of digital twins in the health domain, aiming to synthesize and critically interpret a rapidly expanding, multidisciplinary, and methodologically heterogeneous body of peer-reviewed literature. Given the absence of a universally accepted definition of digital twins in this field, as well as the diversity of methodological approaches spanning clinical, computational, and systems-level studies, a narrative design was considered most appropriate to support structured interpretation and integrative synthesis across domains, rather than a purely aggregative reporting of findings.

The literature search was conducted across three major bibliographic databases: PubMed, Scopus, and Web of Science, selected to ensure comprehensive coverage of high-quality peer-reviewed contributions in biomedical, engineering, and interdisciplinary journals. Only studies with at least the abstract available in English were considered for inclusion.

To maintain methodological rigor and scientific validity, the review included only peer-reviewed journal articles. Conference proceedings, and abstracts were excluded.

In addition, each study included in the synthesis underwent an internal consensus process among the authors to confirm its relevance and methodological suitability for inclusion. A formal record of this consensus process was documented in dedicated minutes and systematically reported in a summary table provided in the Supplementary Materials, along with further details of the selection process, ensuring transparency and traceability of study selection decisions (see Table S1).

The search strategy was designed to identify literature explicitly addressing digital twin concepts in this field, using combinations of the following terms:

(“digital twin*”[Title/Abstract] OR “virtual patient*”[Title/Abstract] OR “in silico patient*”[Title/Abstract] OR “patient specific model*”[Title/Abstract] OR “digital human*”[Title/Abstract] OR “virtual replica*”[Title/Abstract])

To ensure focus on consolidated and higher-level evidence, priority was given to secondary literature already indexed within the databases under systematic review and meta-analysis filters, including systematic reviews, meta-analyses, and umbrella reviews. Within this subset, structured reviews and focused studies were also considered when necessary to capture emerging areas not yet fully addressed by higher-level syntheses in the field of digital twin research.

The literature search and data extraction were conducted during the period of this study. No restrictions were applied regarding year of publication; however, during the selection process, greater consideration was given to recent studies that have updated, expanded, or challenged previous findings, in order to reflect the most current developments in the field.

Studies were included when they explicitly addressed digital twin concepts in healthcare, including patient-, organ-, or system-level digital representations with clinical, translational, preventive, or decision-support relevance, and when they were not purely technical or mathematical in nature, provided they maintained a clear focus on healthcare-oriented digital twin applications. Studies involving educational or training applications were also included when these were directly grounded in digital twin models and linked to clinically relevant use cases such as simulation, procedural planning, surgical rehearsal, rehabilitation, or patient-specific applications. Furthermore, studies with human-centric and ergonomics-related perspectives were also considered when they provided relevant conceptual or methodological contributions to the understanding of digital twin approaches in healthcare.

Study selection followed a concept-driven approach centered on the presence of digital twin frameworks in healthcare contexts. Eligible studies included applications involving patient-, organ-, or system-level digital twins, as well as translational or clinical implementations across different medical domains such as cardiology, neurology, oncology, endocrinology, reproductive medicine, rehabilitation, and healthcare systems.

Given the interdisciplinary nature of the field, studies were not restricted by clinical specialty, allowing inclusion of diverse implementations reflecting the multiple scales and purposes of digital twins in healthcare, from simulation and prediction to monitoring and decision support.

We followed the ANDJ checklist for narrative reviews in structuring the presentation of the findings, ensuring a transparent and well-organized synthesis of the included evidence.

Within the study design, a total of 28 peer-reviewed studies were selected for detailed analysis [21,22,23,24,25,26,27,28,29,30,31,32,33,34,35,36,37,38,39,40,41,42,43,44,45,46,47,48].

## 3. Results

The results are organized into three main sections (Section 3.1, Section 3.2 and Section 3.3), each corresponding to a progressively deeper level of analysis of digital twin (DT) applications in healthcare, ranging from thematic synthesis and conceptual categorization to cross-domain comparison and critical methodological reflection on data, interpretability, ethics, and implementation challenges.

Section 3.1 presents the main emerging themes and the overall categorization of the included studies. The analysis shows a strong convergence in the way Digital Twin technologies are applied across healthcare domains. Four main thematic areas are identified: continuous and data-driven patient representation through multimodal and real-time data integration; predictive and simulation-based medicine using AI and mechanistic models for disease forecasting and treatment optimization; procedural and interventional applications supporting surgery, radiology, and rehabilitation through planning, guidance, and training; and ecosystem-level integration where DTS are embedded within broader digital infrastructures including AI, IoT, and advanced data governance systems. Across all domains, recurrent challenges include interoperability, data quality, validation, and health system integration. Table 1 provides a detailed structured overview of the included studies, summarizing clinical focus, methodologies, key findings, and conceptual contributions, enabling a study-level mapping of evidence into thematic categories. The broad categorization of these themes is further synthesized in Table 2, which provides an overarching grouping of digital twin contributions into higher-level functional domains.

Section 3.2 provides a cross-domain comparative synthesis of the evidence, highlighting a clear gradient of maturity across applications. More advanced implementations are observed in radiology, cardiology, and chronic disease management, where structured data and established clinical workflows support partial integration of DTS into practice. Intermediate maturity characterizes oncology, neurology, and in-silico clinical trials, where DTS are actively used for prediction and simulation but remain limited by validation, explainability, and reproducibility issues. Early-stage or exploratory applications are found in reproductive medicine, blockchain-based health systems, and multi-omics dermatology, where DTS remain largely conceptual or prototype-based. Overall, development is strongly influenced by data availability, infrastructure readiness, and regulatory context, producing a non-linear and heterogeneous trajectory of adoption.

Section 3.3 presents a critical analysis of key limitations and conceptual issues. Section 3.3.1 addresses data complexity and highlights how increasing data volume and heterogeneity can introduce noise, overfitting, and integration problems, particularly in high-dimensional biomedical domains such as genomics and multi-omics. Section 3.3.2 focuses on interpretability and clinical translation, emphasizing that high predictive performance does not necessarily ensure clinical usefulness if models lack transparency, reproducibility, and integration into clinical reasoning. Section 3.3.3 examines ethical, legal, and governance challenges, particularly related to privacy, consent, data ownership, and the lack of standardized regulatory frameworks, with emerging solutions such as blockchain remaining at early stages. Section 3.3.4 clarifies the conceptual positioning of digital twins, distinguishing them from static databases and traditional predictive systems by emphasizing their dynamic, bidirectional integration of data and computational models enabling continuous simulation and updating of patient states.

Overall, the findings indicate that digital twins are evolving toward a unified, multi-layered paradigm in healthcare, characterized by continuous patient modeling, predictive simulation, procedural integration, ecosystem embedding, and system-level transformation, while still facing significant methodological, ethical, and implementation-related constraints.

### 3.1. Emerging Themes and Categorization

The analysis of the included studies reveals a strong conceptual convergence in how digital twin (DT) technologies are framed and applied within healthcare. Despite the diversity of clinical domains, methodologies, and technological implementations, the literature consistently points toward a set of shared underlying principles that define the current trajectory of DTS in medicine.

A first dominant theme is the shift toward continuous, data-driven patient representation, where DTS act as dynamic entities updated through real-time or near real-time data streams. Across chronic disease management contexts, including diet-related conditions, diabetes, and heart failure, these systems integrate wearable and behavioral data to enable adaptive monitoring and personalized feedback loops [21,26,36]. This is further reinforced by pediatric applications, where AI-driven DT frameworks integrate continuous glucose monitoring, closed-loop insulin delivery systems, and telemedicine platforms to support improved glycemic control and earlier risk detection [40]. Together, these findings reflect a broader movement from episodic care to longitudinal, data-intensive patient modeling, with DTS functioning as evolving clinical entities rather than static representations.

Closely related is the emergence of predictive and simulation-based medicine, in which DTS are used not only to represent the current state of a patient but also to anticipate future trajectories. This is particularly evident in oncology and cardiology, where virtual models allow simulation of disease progression and testing of therapeutic strategies in a risk-free environment [22,24,28,37]. Similar predictive extensions are observed in radiology and MRI applications, where DTS support diagnostic prediction, treatment optimisation, and modality-specific planning, particularly in cardiology and oncology imaging workflows [39,44]. DTS also support in-silico clinical trials, enabling virtual experimentation at individual and population levels, improving inclusivity, and optimizing trial design [27]. Across these domains, predictive modeling is increasingly strengthened by AI systems embedded within DT architectures, including self-learning neural networks and multimodal machine learning approaches [40,43].

Another key theme concerns the integration of DTS into interventional and procedural contexts, where they support decision-making before and during clinical actions. In surgical domains—including neurosurgery and plastic surgery—and in robot-assisted interventions, DTS enable preoperative simulation, risk assessment, and procedural optimization [29,31,34]. Radiology and imaging-based DTS further extend this capability to interventional planning, automated dosimetry, and radiographer training, highlighting their role in both diagnostic and procedural workflows [44]. In rehabilitation, advanced digital human models combined with extended reality technologies allow the personalization of therapeutic pathways and functional recovery strategies [30], while human-centric DTS originating from Industry 5.0 frameworks further emphasize ergonomics, task allocation, and human-system interaction [47,48].

A further emerging theme is the development of hybrid technological ecosystems, in which DTS are components of broader digital health infrastructures. These ecosystems incorporate artificial intelligence, advanced analytics, and novel data governance mechanisms, such as blockchain-based approaches and non-fungible tokens (NFTs), to support data ownership, interoperability, and scalability [25,32,33,35]. Similar ecosystem-level transformations are observed in the pharmaceutical sector, where DTS are positioned within value-chain-wide digital transformation processes spanning drug discovery, manufacturing, supply chains, and patient-centric models [41]. In dermatology, AI-driven cosmetogenomics integrates genomic, proteomic, and imaging data with DT frameworks to enable precision-based interventions in personalized skincare [42]. Across these domains, DTS increasingly function as infrastructural components within complex adaptive healthcare ecosystems rather than isolated technological tools.

Finally, several studies converge on the importance of implementation, scalability, and health system integration, emphasizing that the impact of DTS extends beyond individual patient applications. Infrastructure readiness, regulatory frameworks, and applicability in diverse healthcare settings, including low- and middle-income countries, are increasingly recognized as central to successful adoption [25,33,38]. Additional evidence highlights persistent barriers such as data quality, interoperability gaps, unequal technological access, and limited long-term clinical validation, particularly in pediatric care, imaging systems, and pharmaceutical deployment contexts [40,41,44]. Concerns related to equity, governance, and digital divide effects further emphasize that DT adoption is unevenly distributed across institutions, populations, and healthcare systems [41,46]. These studies collectively underline that DT evolution is not only a technological trajectory but a systemic transformation requiring coordinated advancements across policy, clinical practice, and innovation.

Taken together, these themes indicate that DTS are evolving from experimental and domain-specific tools into a unifying paradigm for healthcare innovation, characterized by continuous data integration, predictive modeling, procedural support, and system-level transformation. Across imaging [39,44], pediatric care [40], pharmaceuticals [41], dermatology [42], neurology and headache medicine [43], and human-centric industrial-health interfaces [47,48], a consistent convergence emerges toward AI-augmented, interoperable, and multi-scale digital ecosystems.

Overall, Table 1 reinforces the interpretation that DT technologies are converging toward a multidimensional, integrative paradigm, simultaneously enabling continuous patient modelling, predictive simulation, procedural enhancement, and system-wide innovation. This demonstrates a clear pattern of conceptual convergence across heterogeneous clinical domains, confirming that DTS are moving toward becoming a comprehensive and unifying approach in healthcare.

The analysis of the included studies reveals a strong conceptual convergence regarding the application of digital twin (DT) technologies in healthcare, with six macro-dimensions emerging that collectively describe how DTS are reshaping clinical practice, biomedical research, and health system organization. Despite heterogeneity in clinical domains, methodological designs, and technological maturity, the literature consistently indicates a progressive transition toward integrated, predictive, and system-level digital healthcare architectures.

*A first and dominant dimension concerns continuous, multimodal and personalized patient representation*. Across multiple studies, DTS are conceptualized as dynamic, continuously updated digital constructs integrating physiological signals, wearable device outputs, behavioral information, imaging data, and clinical records to produce evolving patient-specific models. This paradigm is particularly evident in chronic disease management, including diabetes, cardiovascular diseases, diet-related conditions, and pediatric metabolic disorders, where DTS enable longitudinal monitoring, adaptive feedback, early warning systems, and individualized therapeutic adjustments [21,36,40,46]. In these contexts, DTS increasingly move beyond simple monitoring tools toward adaptive systems capable of supporting self-management, improving adherence, and enabling precision prevention strategies.

*A second macro-dimension involves predictive modeling and simulation-based medicine, where DTS are used not only to represent current physiological states but also to simulate disease trajectories and treatment responses under multiple scenarios*. This includes oncology, cardiology, neurology, headache disorders, and other complex conditions, where hybrid computational models, machine learning systems, and mechanistic simulations are used to anticipate progression, optimize interventions, and support clinical decision-making under uncertainty [22,24,28,37,43]. Within this same dimension, the literature highlights the emergence of in-silico clinical trials, enabling virtual experimentation at both individual and population levels, improving efficiency, reducing costs, and potentially increasing inclusivity in study design [27,45]. The same predictive logic is also extended to pharmaceutical pipelines and translational research, where DTS are positioned as accelerators of drug development and systems-level modeling tools [41].

*A third dimension relates to procedural, interventional, and imaging-guided applications, where DTS are embedded into clinical workflows that directly support diagnosis and treatment execution.* In radiology and MRI environments, DTS are used for diagnostic enhancement, protocol optimization, safety assurance, and training, with emerging applications in cardiology and oncology imaging workflows [39,44]. In surgical and interventional domains, including neurosurgery, robotic surgery, and minimally invasive procedures, DTS support preoperative planning, intraoperative navigation, and risk reduction [29,31,34]. Complementarily, rehabilitation contexts integrate DTS with extended reality and digital human models to enable adaptive, patient-specific recovery pathways [30]. Importantly, imaging-focused reviews also highlight underexplored areas such as operational efficiency, environmental sustainability, and training systems, expanding the role of DTS beyond strictly diagnostic functions [39].

*A fourth macro-dimension concerns integration within broader digital health and AI-driven ecosystems, where DTS function as interconnected components within complex socio-technical infrastructures.* Across studies, DTS are increasingly embedded within platforms combining artificial intelligence, advanced analytics, Internet of Things architectures, and emerging governance mechanisms such as blockchain and decentralized data systems. These infrastructures support interoperability, secure data exchange, and scalable computation while also introducing new paradigms of data ownership and patient-centered control [25,32,33,35]. This ecosystem perspective is also reinforced in cosmetogenomics and multi-omics dermatology, where DTS are integrated with genomic and proteomic data to support highly individualized therapeutic strategies [42], and in headache medicine, where DTS contribute to multimodal diagnostic and therapeutic decision-support systems [43].

*A fifth dimension addresses implementation, scalability, regulatory alignment, and health system integration*. Across the literature, a consistent message is that the effectiveness of DTS depends not only on technological maturity but also on infrastructure readiness, governance frameworks, interoperability standards, and ethical oversight. These challenges are particularly relevant in diverse healthcare environments, including low- and middle-income countries, where disparities in access, resources, and digital maturity significantly influence adoption [25,33,38,45]. In this sense, DT implementation is increasingly framed as a systemic transformation requiring alignment between technological innovation, institutional capacity, and policy frameworks.

*A sixth and cross-cutting dimension encompasses domain diversification, human-centric design, and socio-technical transformation.* Here, DT applications extend beyond traditional clinical domains into pediatric endocrinology, pharmaceuticals, personalized dermatology, industrial and ergonomic systems, and human-centric modeling environments [40,41,42,47,48]. In pediatric diabetes care, DTS are integrated with AI-driven predictive systems and closed-loop therapeutic technologies to enhance glycemic control and autonomy [40]. In industrial and ergonomic contexts, human-centric DTS support simulation of human performance, optimization of workflows, and integration with cyber-physical systems [47,48]. Across these domains, a recurring emphasis is placed on safety, training, operational optimization, sustainability, and workforce interaction, indicating that DTS are not only clinical tools but also broader socio-technical infrastructures shaping human-environment interaction. Collectively, these six dimensions illustrate that digital twins are evolving from fragmented, domain-specific innovations into a unified, multi-layered paradigm characterized by continuous personalization, predictive simulation, procedural integration, ecosystem embedding, systemic scalability, and cross-domain socio-technical expansion.

These dimensions are synthesized in Table 2, which provides a structured overview of the main application areas and their corresponding contributions. Notably, several studies contribute to more than one macro-dimension, reflecting the inherently transversal and multi-layered nature of digital twin research. As a result, partial overlaps in classification are expected and intentionally preserved, since they reflect conceptual intersections between clinical applications, technological infrastructures, and system-level healthcare transformation rather than methodological redundancy. Collectively, these six dimensions illustrate that digital twins are evolving from fragmented, domain-specific innovations into a unified, multi-layered paradigm characterized by continuous personalization, predictive simulation, procedural integration, ecosystem embedding, systemic scalability, and cross-domain socio-technical expansion.

### 3.2. Cross-Domain Comparative Synthesis and Evidence Maturity Gradient

Across the included literature, a cross-domain comparative synthesis of digital twin (DT) applications in healthcare reveals a consistent but non-uniform pattern of development in terms of technological maturity, methodological robustness, and translational readiness. Rather than progressing uniformly across clinical fields, DT research appears distributed along a stratified continuum in which different domains occupy distinct positions depending on data availability, model complexity, validation practices, and integration within clinical workflows.

At one end of this continuum, relatively more mature implementations are observed in radiology, cardiology, and chronic disease management. In these domains, DT development benefits from structured and high-resolution data ecosystems, including imaging pipelines, electronic health records, and continuous physiological monitoring systems. For example, radiology-based DT frameworks leverage multimodal imaging data and AI-assisted reconstruction pipelines to support diagnostic enhancement, treatment planning, dosimetry optimization, and workflow efficiency [39,44]. Similarly, in cardiology and cardiovascular modeling, DTS integrate ECG signals, imaging data, and mechanistic or hybrid AI models to support risk stratification, therapy planning, and disease monitoring, although clinical validation and real-world outcome evidence remain partially limited [24,37,46]. Chronic disease applications, particularly diabetes and heart failure, further demonstrate the integration of wearable technologies and continuous monitoring systems into longitudinal patient models, enabling adaptive feedback and personalized disease management strategies [21,36,40]. In these contexts, DTS increasingly operate as dynamic monitoring-support systems embedded within existing care pathways rather than fully autonomous clinical infrastructures.

In contrast, several emerging domains remain predominantly at exploratory or early validation stages. Reproductive medicine, for instance, relies heavily on simulation-based DT models for embryo selection, pregnancy modeling, and pharmacokinetic simulations, but these approaches are still largely theoretical and characterized by limited clinical validation and relatively high methodological uncertainty [23]. Similarly, blockchain- and NFT-based DT frameworks focus primarily on conceptual architectures for decentralized health data governance, patient ownership, and traceability, with current evidence largely restricted to prototypes and early-stage implementations rather than large-scale clinical deployment [32]. In dermatology and cosmetogenomics, AI- and multi-omics-driven DT approaches show strong potential for precision personalization, yet remain constrained by heterogeneous endpoints, limited population diversity, and the absence of standardized validation frameworks [42]. Across these domains, DTS are best understood as evolving conceptual and computational frameworks rather than fully operational clinical systems.

Between these two poles lies a broad intermediate space where digital twins are actively transitioning from simulation-based prototypes to partially validated clinical tools. This is particularly evident in oncology, neurology, and headache medicine, where AI-driven DT frameworks are used to model disease progression, predict treatment response, and support clinical decision-making under uncertainty [22,28,43]. However, across these applications, persistent limitations remain regarding external validation, explainability, reproducibility, and integration into real-world clinical workflows. Cardiac in-silico clinical trials further illustrate this intermediate stage, showing promising applications in virtual experimentation and intervention testing, while simultaneously highlighting gaps in standardization, uncertainty quantification, and reporting practices [37].

From a methodological perspective, the included studies reflect substantial heterogeneity in design, analytical approaches, and levels of evidence synthesis. The corpus includes systematic reviews, umbrella reviews, scoping reviews, meta-analyses, structural topic modeling, and qualitative syntheses, reflecting both the interdisciplinary nature of DT research and its early developmental stage as a field. While this methodological diversity enables broad conceptual coverage, it also limits direct comparability across studies and contributes to variability in reported outcomes, levels of validation, and translational claims. For instance, reviews focusing on system-level DT adoption emphasize infrastructural and governance challenges [25,45], whereas domain-specific clinical reviews tend to emphasize predictive performance and technical feasibility [22,24,28,44].

A key finding emerging from this cross-domain comparison is the presence of an evidence maturity gradient rather than a uniform level of technological readiness. At the lower end of this gradient, DTS function primarily as conceptual or theoretical models used to explore potential applications and system architectures. At intermediate levels, they operate as computational prototypes validated on retrospective datasets or limited clinical cohorts. At higher levels of maturity, particularly in imaging, chronic disease monitoring, and selected surgical applications, DTS are partially integrated into clinical workflows, supporting decision-making, planning, and training, although long-term outcome evidence remains limited [29,30,31,34,39,44].

Importantly, this gradient is shaped not only by computational advances but also by systemic and contextual factors. The availability of structured data, the maturity of healthcare infrastructures, regulatory clarity, and the presence of standardized clinical protocols all appear to significantly influence the pace of DT development. Domains with established measurement systems and well-defined clinical endpoints, such as radiology and cardiology, tend to exhibit higher levels of DT maturity. Conversely, fields characterized by biological complexity, heterogeneous data sources, or emerging digital infrastructures, such as reproductive medicine and blockchain-based systems, remain at earlier stages of development.

In addition, several studies highlight that digital divide effects and disparities in healthcare infrastructure contribute to uneven adoption patterns across regions and institutions. Low- and middle-income healthcare systems are frequently underrepresented in advanced DT implementations, reflecting broader structural inequalities in access to computational resources, data infrastructures, and digital health governance frameworks [38,41,45]. This reinforces the interpretation that DT maturity is not solely a technological phenomenon but also a reflection of broader socio-economic and institutional conditions.

Taken together, the cross-domain synthesis suggests that digital twin development in healthcare should be understood as a heterogeneous and multi-layered trajectory rather than a linear progression toward a single end-point. While certain domains demonstrate relatively advanced integration into clinical workflows, others remain in exploratory or prototype stages, and many occupy intermediate positions characterized by partial validation and limited real-world deployment. This heterogeneity is not incidental but reflects the inherently interdisciplinary nature of DT research and the varying degrees of readiness across clinical, technological, and institutional contexts.

Overall, the evidence supports the interpretation that digital twins are emerging as a transformative but unevenly developed paradigm in healthcare. Their evolution is shaped by the interaction between data availability [21,36,40], computational modeling approaches [22,28,37], imaging and procedural integration [39,44], ecosystem-level infrastructures [25,32,45], and system-wide adoption constraints [38,41]. Recognizing this stratified maturity landscape is essential for accurately interpreting current evidence, avoiding overgeneralized claims, and guiding future research toward domains where translational gaps remain most pronounced.

### 3.3. Critical Reflections on Data, AI, and Digital Twin Implementation

The rapid expansion of digital health technologies, including artificial intelligence (AI), large-scale data collection systems, and digital twin (DT) frameworks, has generated substantial enthusiasm regarding their potential to transform healthcare delivery, research, and clinical decision-making. In particular, the increasing availability of multimodal data sources—ranging from wearable devices and electronic health records to genomic, imaging, behavioral, and pharmacological datasets—has enabled the development of increasingly complex predictive and simulation-based models. Within this context, digital twins are often positioned as integrative constructs capable of combining heterogeneous data streams into dynamic, continuously updated patient-specific representations that support precision medicine and individualized care pathways across chronic diseases, oncology, cardiology, neurology, pediatrics, and systemic healthcare applications [21,22,24,28,40,44,46].

However, alongside these advances, important conceptual and methodological challenges emerge when moving from data accumulation to clinically meaningful inference. The assumption that increasing data volume, feature dimensionality, and algorithmic complexity necessarily translates into improved clinical insight remains contested. In several biomedical domains, particularly those involving high-dimensional data such as genomics, multi-omics profiling, and single-nucleotide polymorphism (SNP) analysis, issues related to noise accumulation, overfitting, bias propagation, and limited external validity have been widely documented. These limitations are reflected in studies where predictive performance is high but reproducibility, multimodal integration, and real-world generalizability remain constrained [22,42]. Similarly, disease-specific DT models, such as those in type 1 diabetes and cardiology, highlight that current systems often remain incomplete or partially validated representations of physiological reality rather than fully integrated multiscale replicas [24,26].

Moreover, the transition from data-rich environments to clinically actionable knowledge requires careful consideration of statistical validity, model robustness, and biological interpretability. In AI-driven healthcare systems, predictive performance alone does not necessarily correspond to clinical utility, decision transparency, or causal understanding. This is particularly evident in domains such as neuro-oncology and prostate cancer care, where AI-based DTS demonstrate high predictive accuracy but still face limitations related to explainability, real-time integration, and external clinical validation [22,28]. Similar concerns are echoed in cardiac in-silico clinical trials, where variability in validation practices, incomplete reporting, and limited model standardization restrict translational reliability [37]. Across radiology and imaging-based DT systems, computational complexity and insufficient clinical validation further reinforce these interpretability and deployment gaps [39,44].

In parallel, the expansion of data-driven healthcare ecosystems raises critical ethical, legal, and societal considerations. Continuous data acquisition through wearable technologies, IoT-enabled monitoring systems, and interconnected clinical infrastructures introduces complex challenges related to privacy, informed consent, data ownership, and long-term governance. These issues are particularly evident in studies integrating wearable devices and edge AI for chronic disease management, where privacy-preserving monitoring is proposed but interoperability and architectural standardization remain unresolved [36]. Similarly, blockchain- and NFT-enabled frameworks for patient data control highlight both the potential for decentralized governance and the persistence of technological, economic, and interoperability barriers [32]. Across broader healthcare systems, umbrella reviews emphasize persistent concerns regarding data privacy, high implementation costs, and ethical governance requirements as central constraints to scalable adoption [25,45].

Furthermore, the conceptual framing of “digital twins” itself requires clarification within the broader landscape of healthcare digitalization. In several applications, DTS overlap partially with traditional electronic health records, longitudinal datasets, predictive analytics systems, and AI-based decision support tools, raising questions about their incremental conceptual and functional value. This ambiguity is reflected across multiple domains, including pediatric diabetes, cardiology, and general healthcare systems, where DTS are sometimes implemented as extensions of predictive modeling rather than fully bidirectional, continuously updated virtual representations [24,40,45]. In contrast, more advanced implementations emphasize closed-loop systems integrating simulation, prediction, and feedback mechanisms, thereby distinguishing DTS from conventional data aggregation approaches [21,22]. Establishing this conceptual boundary is essential to avoid terminological inflation and to ensure analytical rigor across clinical and research applications.

To address these issues, the following subsections critically examine key dimensions underlying data-driven AI and digital twin implementation in healthcare, focusing on: (i) data complexity and analytical limitations, (ii) interpretability and clinical translation, (iii) ethical and governance challenges, and (iv) the conceptual positioning of digital twins within modern healthcare systems.

#### 3.3.1. Data Complexity and Analytical Limitations

The growing adoption of digital health infrastructures and AI-driven digital twin (DT) systems is strongly associated with an unprecedented increase in the volume, heterogeneity, and granularity of patient-related data. Across the included studies, this expansion is consistently observed through the integration of wearable sensors, electronic health records, imaging data, and multi-omics information into unified modeling frameworks [21,22,23,24,25,40,42]. Importantly, this same expansion simultaneously introduces both methodological and governance-related constraints, indicating that data growth cannot be interpreted as a purely positive or neutral development.

In particular, several studies emphasize that high-dimensional biomedical datasets introduce substantial analytical challenges, including noise accumulation, overfitting, and limited model generalizability, especially when the number of variables far exceeds the number of patients or clinically independent observations [22,24,28,37]. These limitations are well illustrated in complex domains such as genomics and molecular profiling, where large-scale feature spaces (e.g., SNP-based or multi-omic datasets) may improve predictive performance in controlled settings but often reduce interpretability, stability, and external validity in real-world clinical environments. Similar concerns emerge in AI-driven DT applications in oncology and cardiology, where performance is frequently constrained by data quality, heterogeneous acquisition protocols, and insufficient external validation [22,24,28,39].

Crucially, this data expansion also raises a structural tension between predictive enrichment and informational degradation. The increasing reliance on multi-source, continuously generated datasets (e.g., wearable technologies, remote monitoring systems, and behavioral tracking platforms) introduces additional layers of variability, potential oversampling, and non-independent observations, which may amplify statistical noise rather than reduce uncertainty. In this sense, more data does not necessarily equate to more knowledge, particularly when data streams are not harmonized or clinically contextualized.

Alongside these methodological constraints, the expansion of data-intensive DT systems introduces a parallel and equally critical dimension related to patient privacy, confidentiality, and rights protection. Continuous data acquisition from wearable devices, IoT-enabled monitoring systems, and interconnected clinical infrastructures generates persistent challenges regarding informed consent, data ownership, secondary use of data, and long-term governance responsibilities. Several studies explicitly highlight that privacy is not a secondary technical requirement but a core condition for system legitimacy, patient trust, and sustainable adoption of DT-based healthcare solutions [25,27,32,45]. However, the literature also shows that privacy-preserving architectures and patient-centric rights frameworks remain unevenly implemented, often lagging behind technological innovation.

More broadly, umbrella and meta-review evidence indicates that fragmentation of data ecosystems and the absence of standardized governance models remain persistent barriers to large-scale DT implementation [25,45]. These structural limitations are particularly relevant in scenarios requiring cross-institutional data sharing and integration of heterogeneous data modalities, where the tension between data utility and patient protection becomes especially pronounced.

Finally, clarification of the conceptual positioning of digital twins is essential to avoid terminological ambiguity. While DTS are often described as integrative systems combining longitudinal patient data and predictive analytics, several studies emphasize that their added value lies not in data aggregation per se, but in the continuous coupling of data with dynamic computational models capable of simulation, prediction, and scenario testing [25,33,45]. In this sense, DTS should not be interpreted as extensions of electronic health records, but rather as model-driven systems enabling iterative, bidirectional interaction between patient data and computational representations.

Collectively, these findings suggest that the effectiveness of AI and DT frameworks in healthcare depends less on data volume alone and more on the quality of integration, interpretability of models, and robustness of ethical governance structures, including privacy, consent, and patient rights protections.

#### 3.3.2. Interpretability, Clinical Translation, and the Limits of Data-Centric AI in Digital Twins

Beyond the challenges related to data complexity, an additional critical issue concerns the relationship between data intensity, model performance, and clinical interpretability in digital twin (DT) and AI-driven healthcare systems. A recurrent assumption in the literature is that increasing the number of data sources—such as wearable devices, electronic health records, imaging repositories, and multi-omics datasets—automatically leads to improved predictive accuracy and clinical insight. However, several studies included in this review challenge this premise by showing that more data can also introduce additional variability, noise, and redundancy, potentially reducing model robustness and interpretability rather than enhancing it [22,24,27,37].

This issue is particularly evident in high-dimensional biomedical domains, such as genomics and precision oncology, where the number of features may far exceed the number of clinically independent observations. In neuro-oncology and prostate cancer applications, for example, AI-driven DTS demonstrate high predictive performance in controlled settings but remain limited by external validation constraints, reproducibility issues, and insufficient model transparency [22,28]. Similarly, in cardiac in-silico clinical trials, variability in modeling assumptions and incomplete reporting of computational pipelines further complicate the translation of data-driven outputs into reliable clinical evidence [37]. These findings collectively support the view that increasing data dimensionality does not inherently resolve uncertainty in clinical decision-making and may, in some cases, amplify methodological fragility.

Within this context, concerns related to oversampling, dataset bias, and unbalanced population representation further complicate the development of reliable DT systems. As highlighted in studies focusing on clinical trial design and digital health equity, AI-based DT frameworks may inadvertently reinforce existing biases when trained on non-representative datasets, limiting their generalizability across demographic groups and clinical settings [27,41]. This reinforces the need to move beyond purely data-centric paradigms toward hybrid approaches that combine mechanistic modeling, clinical expertise, and statistically robust validation frameworks.

A related and equally important concern raised in the literature is the risk that AI-driven healthcare systems may bypass traditional clinical reasoning by overemphasizing automated pattern recognition. Importantly, within the digital twin paradigm, the primary objective is not the maximization of predictive accuracy through data accumulation, but the preservation of clinically meaningful interpretability and mechanistic coherence that allows model outputs to remain embedded within established medical reasoning and decision-making frameworks.

While DTS are frequently positioned as tools for enhancing decision-making, several studies emphasize that predictive performance alone is insufficient if not accompanied by clinical interpretability, causal plausibility, and actionable insights [24,25,43]. In precision cardiology and headache medicine, for instance, the integration of AI and DT approaches improves risk stratification and diagnostic support, yet persistent limitations remain in explainability and clinical integration within real-world workflows [24,43].

Finally, the conceptual distinction between digital twins and conventional electronic health records or longitudinal patient databases requires careful clarification. Several contributions in this review indicate that DTS should not be interpreted as a simple rebranding of large-scale patient data collection systems. Instead, their defining feature lies in the continuous coupling between patient-specific data and dynamic computational models capable of simulation, prediction, and iterative updating [25,33,45]. From this perspective, the added value of DTS is not merely the accumulation of information, but the transformation of heterogeneous data streams into executable, model-driven representations of physiological and pathological processes. Studies in healthcare system-level DTS and imaging-based applications reinforce this view by highlighting the importance of integration, simulation capability, and feedback mechanisms as key differentiators from traditional data repositories [39,45].

Overall, the evidence suggests that while data availability and AI techniques are essential enablers of digital twin systems, their clinical value depends critically on interpretability, validation rigor, and the ability to integrate computational outputs with established medical reasoning rather than replacing it.

#### 3.3.3. Ethical, Legal, and Governance Challenges in Data-Driven and Digital Twin Healthcare

Alongside methodological and interpretative limitations, the expansion of AI-driven healthcare systems and digital twin (DT) frameworks raises substantial ethical, legal, and governance challenges that are increasingly recognized across the literature. The continuous acquisition of patient-generated data through wearable devices, connected health platforms, and clinical information systems introduces complex issues related to privacy protection, data confidentiality, informed consent, data ownership, and long-term stewardship of sensitive health information. Several studies included in this review consistently emphasize that these concerns are not peripheral, but instead constitute structural requirements for the safe and sustainable deployment of DT-based healthcare ecosystems [25,32,45].

In particular, emerging governance approaches attempt to respond to these challenges by rethinking how patient data are stored, accessed, and shared across distributed healthcare systems. Blockchain-based architectures and NFT-enabled frameworks have been proposed as mechanisms to enhance traceability, transparency, and patient control over data flows, enabling more granular management of consent and data provenance [32]. However, the literature also highlights that such approaches remain largely at the prototype or early validation stage, with unresolved issues related to scalability, interoperability with existing hospital infrastructures, and regulatory acceptance.

More broadly, umbrella and meta-review evidence indicates that fragmentation of data ecosystems and the absence of standardized governance models represent persistent barriers to the implementation of DT systems at scale [25,45]. These structural limitations are particularly relevant when considering cross-institutional data sharing and integration of heterogeneous sources such as imaging, genomic, wearable, and electronic health record data. Without robust governance frameworks, the potential of DTS to enable continuous learning systems in healthcare remains partially constrained.

Importantly, several clinical-domain studies further demonstrate that ethical concerns are closely interwoven with methodological issues such as bias, representativeness, and model fairness. In applications involving AI-enhanced clinical decision support and predictive modeling, including neurology, cardiology, and population-level precision health, concerns about dataset imbalance and privacy-preserving data use directly affect both performance and equity of outcomes [27,43,46]. In this sense, ethical governance cannot be treated as an external layer added after technical development, but must be integrated within the design of DT systems from the earliest stages.

Overall, the reviewed literature converges on the view that while digital twins offer significant opportunities for enhancing personalized and predictive healthcare, their real-world deployment depends on the development of robust ethical frameworks, clear regulatory pathways, and interoperable governance infrastructures capable of ensuring patient rights, data security, algorithmic accountability, and responsible innovation at system scale.

#### 3.3.4. Conceptual Positioning of Digital Twins: From Data Aggregation to Dynamic Simulation Systems

A further key issue raised in the literature concerns the conceptual definition and epistemological positioning of digital twins within healthcare, particularly in response to critiques suggesting that DTS may represent little more than a rebranding of existing electronic health records or large-scale patient data repositories. Addressing this concern, the reviewed studies consistently indicate that the defining feature of digital twins lies not in the mere aggregation of data, but in the continuous and bidirectional interaction between multimodal data streams and computational models capable of simulation, prediction, and iterative updating of patient-specific states [25,33,45].

Importantly, the added value of the “digital twin” concept lies in its transition from purely descriptive data representation toward a dynamic, model-driven paradigm in which patient-specific data are continuously integrated with computational models to enable simulation, prediction, and intervention testing under varying clinical scenarios, thereby moving beyond static longitudinal datasets toward actionable and experimentally exploitable digital representations of disease processes.

From this perspective, conventional clinical databases and longitudinal health records primarily function as static or retrospective representations of patient information, whereas digital twins introduce a dynamic modeling layer that enables scenario simulation, hypothesis testing, and personalized forecasting under varying physiological or therapeutic conditions. This distinction becomes particularly evident in complex clinical domains such as precision cardiology, neuro-oncology, and in-silico clinical trials, where DT frameworks are explicitly designed to model disease progression trajectories, therapeutic responses, and intervention outcomes in individualized settings [22,24,28,37].

Furthermore, system-level analyses highlight that digital twins should be understood as integrative constructs embedded within broader digital health ecosystems rather than isolated computational tools. Across multiple studies, DTS are described as components of interconnected infrastructures combining artificial intelligence, Internet of Things devices, advanced imaging pipelines, and data governance layers into unified simulation environments that support both clinical and operational decision-making [39,44,45]. In this context, their value emerges from the orchestration of heterogeneous technologies rather than from any single data source or algorithmic component.

Additional evidence from domain-specific applications reinforces this interpretation. In rehabilitation, surgery, radiology, and chronic disease management, digital twins are increasingly used to bridge physiological modeling with real-time data inputs, enabling adaptive interventions, improved procedural planning, and personalized treatment pathways [30,31,34,36]. In radiology and imaging-intensive applications, for example, DTS are not limited to diagnostic support but extend to workflow optimization, training, and system-level efficiency improvements, further emphasizing their multi-layered functionality [39,44].

Taken together, these findings support a clear conceptual distinction: digital twins are not reducible to large-scale data aggregation systems, but instead represent evolving cyber-physical and computational representations of patients and healthcare processes. While challenges remain regarding validation, standardization, and clinical translation, the convergence of evidence across multiple domains supports the interpretation of DTS as a distinct paradigm of data–model integration, in which the primary innovation lies in transforming heterogeneous data into executable, continuously updated simulation systems capable of informing both individual care and population-level healthcare strategies.

## 4. Discussion

This section is organized into seven interconnected subsections. The overall aim of this section is to move from evidence synthesis to interpretative integration, progressively linking structured secondary evidence, empirical findings, conceptual contributions, and policy-level perspectives. The final goal is to construct a coherent analytical framework for understanding the current state, limitations, and future trajectory of digital twin (DT) technologies in healthcare.

Section 4.1 focuses on the synthesis of structured secondary evidence, primarily systematic reviews and meta-analyses. Its purpose is to establish a high-level evidence baseline by identifying recurring cross-domain patterns in the literature. This section shows that digital twins are increasingly conceptualized as dynamic, multi-layered systems that integrate clinical, biological, and computational data streams. At the same time, it highlights a critical tension: while conceptual maturity is rapidly advancing, methodological standardization, reproducibility, and real-world validation remain limited. This section therefore defines both the conceptual consolidation and the structural fragility of the field.

Section 4.2 translates the synthesized evidence into actionable recommendations. Rather than presenting isolated observations, it organizes the findings into six transversal domains (R1–R6), which represent the strategic pillars for advancing the field. These include expansion of clinical scope, methodological standardization, integration of emerging technologies, ethical and governance frameworks, longitudinal validation, and interoperability. The logic of this section is transformative: it converts descriptive evidence into a structured roadmap for research and implementation.

Section 4.3 provides a comparative synthesis across clinical trials, empirical studies, conceptual contributions and integrative contributions. The objective is to move from aggregated evidence to functional interpretation of how digital twins are actually implemented in healthcare systems. Clinical trials demonstrate early but tangible evidence of effectiveness in domains such as diabetes, oncology, and metabolic diseases. Primary and conceptual studies extend this scope into emerging fields such as neuroscience, infectious diseases, pharmacology, and emergency care. This section shows that digital twins are not a single technology but a heterogeneous ecosystem of applications with varying degrees of maturity. Importantly, it also reinforces recurring gaps in validation, scalability, and methodological consistency.

Building on the previous section, which translates the synthesized evidence into actionable recommendations organized across six transversal domains (R1–R6), this section adopts these domains as the interpretative lens for comparative analysis. In this way, the recommendation framework is not merely descriptive, but functions as a structured analytical key for reading heterogeneity, convergence, and divergence across studies.

Section 4.4 situates digital twin development within the broader international policy and governance landscape. The aim is to connect technological and clinical evolution with regulatory and institutional frameworks. This section highlights how organizations such as the FDA and European initiatives like the Virtual Human Twins program are shaping foundational definitions, governance principles, and infrastructure requirements. It also emphasizes the role of international consensus statements in aligning ethical, legal, and interoperability standards. The key message is that digital twins are not developing in isolation but within an increasingly structured global governance ecosystem that is still in formation.

Section 4.5 explains and justifies the methodological choice of a narrative review design. The central argument is that the heterogeneity of the evidence base—spanning clinical trials, computational models, engineering frameworks, and policy documents—cannot be adequately captured through purely systematic or scoping approaches. Instead, a narrative and integrative synthesis is required to preserve conceptual depth while enabling cross-domain interpretation. This section also describes the analytical strategy adopted in the study: recommendations derived from secondary evidence are used as an interpretative framework to analyze primary studies and policy-level contributions, creating a bidirectional synthesis between evidence generation and conceptual structuring.

Section 4.6 identifies future directions for digital twin research in healthcare. It builds directly on the limitations and gaps identified in earlier sections and translates them into forward-looking priorities. These include expanding applications to underrepresented diseases and populations, strengthening interoperability and standardization, integrating emerging technologies such as AI and multi-omics, conducting longitudinal real-world validation studies, and ensuring alignment with evolving regulatory frameworks. The logic here is explicitly developmental: it maps the transition pathway from experimental systems to clinically robust and scalable infrastructures.

Section 4.7 addresses the limitations of the review. It acknowledges the reliance on a targeted PubMed search focusing on structured secondary evidence, which may limit the comprehensiveness of the dataset. However, it also explains how this limitation is partially mitigated through the inclusion of primary studies, randomized trials, and policy documents, which enrich the interpretative depth of the synthesis. Rather than weakening the study, this methodological framing reinforces its integrative nature by combining rigor in evidence selection with breadth in conceptual coverage.

Overall, the Section 4 builds a progressive analytical trajectory: it begins with evidence consolidation (Section 4.1), moves toward structured interpretation and recommendation building (Section 4.2), expands into multi-level empirical validation (Section 4.3), situates the field within global governance structures (Section 4.4), justifies the methodological architecture (Section 4.5), projects future development pathways (Section 4.6), and critically reflects on methodological constraints (Section 4.7). Taken together, these sections demonstrate that digital twins in healthcare are transitioning from fragmented experimental applications toward an integrated socio-technical paradigm. However, their full clinical and systemic adoption depends on resolving persistent challenges in standardization, interoperability, validation, and governance, while strengthening evidence from real-world implementation.

### 4.1. Highlights from the Analysis of Structured Secondary Evidence (Primarily Systematic Reviews and Meta-Analyses)

This section synthesizes findings derived from the analysis of structured secondary evidence, primarily systematic reviews and meta-analyses, focusing on digital twin (DT) applications in healthcare. Rather than presenting original empirical contributions, this synthesis aims to consolidate existing high-level evidence to identify recurring analytical patterns, thematic convergences, and persistent structural limitations across domains.

The reviewed secondary literature consistently indicates that digital twin research in healthcare extends across multiple clinical domains, including diabetes, oncology, cardiology, neurosurgery, radiology, reproductive medicine, and health system optimization [21,22,23,24,25,29,34,36,44]. Selected human-centric and cross-domain applications, including ergonomics and adjacent human factors domains, are also represented when contributing to healthcare-oriented digital twin development.

Across these domains, DTS are increasingly conceptualized as multidimensional systems capable of integrating patient-level, procedure-level, and system-level representations within interconnected healthcare ecosystems.

From a historical perspective, several secondary studies highlight that the conceptual foundations of digital twinning in medicine are not recent. Early forms of patient-specific anatomical and physiological modeling, including 3D vascular reconstructions and simulation-based phantoms, already demonstrated the feasibility of individualized digital representations for clinical planning and procedural simulation [49]. This evidence supports the interpretation of DTS as an evolutionary extension of biomedical modeling rather than an abrupt technological discontinuity.

Across the secondary literature, a consistent finding is that contemporary DT systems differ substantially from traditional health data infrastructures. Rather than functioning solely as data repositories, DTS are increasingly described as dynamic environments integrating artificial intelligence, mechanistic modeling, wearable technologies, imaging systems, and real-time data streams into continuously updated computational representations [21,22,24,40,44]. This evolution is particularly evident in domains such as neuro-oncology, cardiology, and diabetes management, where DTS are used not only for data storage but also for simulation of disease trajectories, therapeutic response, and personalized intervention pathways [22,24,26,28,40].

Another recurrent synthesis emerging from systematic and meta-analytic evidence is the characterization of DTS as integrative systems operating at the interface between individual patient care and healthcare system infrastructure. Secondary studies consistently report their potential to support both individualized clinical decision-making and broader system-level optimization, including surgical planning, rehabilitation pathways, chronic disease management, and healthcare workflow efficiency [27,30,31,34,36]. In addition, emerging digital architectures, including decentralized frameworks and data governance models such as blockchain-based systems, are increasingly discussed as potential enablers of secure and patient-centered DT ecosystems [32,41].

However, structured secondary evidence also consistently highlights significant methodological and translational limitations. These include heterogeneous validation strategies, limited reproducibility, lack of standardized computational frameworks, and insufficient real-world clinical deployment [22,24,37,44]. Despite rapid technological advancement, many DT applications remain at experimental or early translational stages, particularly in complex and high-burden domains such as oncology, cardiology, and reproductive medicine [23,28].

A further key synthesis concerns the conceptual shift introduced by DTS in healthcare. Secondary literature emphasizes that their value does not lie merely in extending electronic health records or predictive analytics systems, but in enabling a transition from static data representation to dynamic, model-driven simulation environments capable of iterative updating, scenario testing, and predictive intervention modeling. This shift is consistently identified as a fundamental distinguishing feature of DTS compared to traditional digital health infrastructures [21,25,33,45].

Overall, the analysis of structured secondary evidence suggests that digital twins are evolving toward integrated cyber-physical and computational systems that bridge individualized medicine and healthcare system intelligence. At the same time, the literature highlights persistent fragmentation, limited standardization, and unresolved translational barriers that currently constrain their maturity and large-scale clinical adoption.

### 4.2. Emerging Recommendations from Integrated Evidence Synthesis

Recommendations emerging from this synthesis are derived from a structured integration of secondary evidence (primarily systematic reviews and meta-analyses), primary studies, real-world implementations, and policy-level documents. Rather than representing isolated findings, these recommendations reflect recurring cross-domain patterns observed across heterogeneous clinical, technological, and organizational contexts. This approach enables the translation of fragmented evidence into higher-order, actionable directions for the development and implementation of digital twin systems in healthcare.

In this framework, recommendations are not interpreted as simple thematic summaries, but as convergent analytical outputs emerging from the interaction between methodological limitations, technological opportunities, and clinical translation gaps identified across the literature. This allows the formulation of a coherent set of transversal domains (R1–R6), which capture the key strategic priorities for advancing digital twin research and deployment in healthcare systems.

**Recommendations emerging from this synthesis include:**

***R1. Broader disease coverage and population diversity: Expand digital twin applications beyond currently dominant domains such as diabetes, cardiology, and neuro-oncology toward multimorbidity, reproductive health, radiology ecosystems, and population-level healthcare systems* [21,22,23,24,25,26,38,44]**.

***R2. Standardization and reproducibility: Develop internationally agreed frameworks for model development, reporting, validation, and open science practices, including code and dataset sharing to improve reproducibility and comparability* [22,24,34,37]**.

***R3. Integration of emerging technologies: Strengthen convergence between artificial intelligence, wearable systems, omics technologies, edge computing, and immersive environments to support real-time and adaptive digital twin ecosystems* [35,36]**.

***R4. Ethical and governance frameworks: Establish robust, scalable, and regulation-aligned frameworks for data ownership, privacy, consent, security, and responsible data monetization in DT-based healthcare systems* [25,32,45]**.

***R5. Longitudinal and real-world validation: Prioritize prospective, multi-center, and real-world clinical studies that move beyond simulation-based evidence toward outcome-driven evaluation of effectiveness and safety* [21,23,34,38]**.

***R6. Interoperability and scalability: Address structural barriers related to cross-platform integration, semantic interoperability, and scalable deployment across heterogeneous healthcare infrastructures* [32,35,44]**.

In summary, digital twins represent a rapidly evolving but still maturing paradigm in healthcare. While technological advances and early clinical results are promising, substantial gaps remain in methodological standardization, ethical governance, interoperability, and real-world validation. Addressing these limitations is essential to bridge the gap between experimental promise and clinically reliable, scalable healthcare implementation.

### 4.3. Cross-Level Recommendation-Guided Synthesis of Digital Twin Evidence in Healthcare (R1–R6)

This section reframes the analysis of digital twin (DT) implementations in healthcare using the transversal recommendation framework (R1–R6) introduced in Section 4.2. The objective is to move beyond author-by-author reporting and to provide a structured comparative interpretation of how different studies contribute to shared dimensions of methodological, clinical, technological, and governance development.

Accordingly, the synthesis integrates evidence from clinical trials, randomized controlled trials (RCTs), primary studies, and non-structured secondary literature. Rather than being treated as independent narratives, these sources are considered functionally distinct but complementary evidence layers within a unified analytical framework. Each contribution is evaluated in terms of its alignment with the six recommendation domains (R1–R6), enabling systematic identification of convergences, gaps, and asymmetries across heterogeneous evidence types.

This approach enables a shift from descriptive synthesis to structured comparative mapping, addressing methodological heterogeneity while preserving interpretative depth.

#### 4.3.1. Clinical Trial Evidence and Its Role in Shaping Digital Twin Recommendations

Clinical trial evidence is analyzed here through the interpretative framework defined by the six transversal recommendations (R1–R6), rather than as isolated demonstrations of technological efficacy. In this perspective, clinical studies are not interpreted solely in terms of outcomes, but as empirical anchors that illuminate how digital twin (DT) systems progressively operationalize broader methodological, technological, and clinical priorities.

In type 1 diabetes, Builes-Montaño et al. [50] demonstrated the STUDIA system, a digital twin-enabled decision support tool that guides insulin dosing. Patients using the system spent more time within the target glucose range and experienced fewer hypoglycemic events. Beyond the clear clinical benefits, this study illuminates the path toward R1, by extending digital twin applications to chronic metabolic conditions, and R2, by showing a reproducible framework others can adopt. The integration of real-time glucose monitoring with AI-driven simulations highlights R3, demonstrating the power of combining data streams and predictive models for personalized interventions.

Shifting to oncology, Saad et al. [51] tackled the challenge of patient selection for combined immunotherapy and stereotactic radiotherapy in early-stage non-small cell lung cancer. By developing a causal AI model that integrates radiomic and clinical data, the study identifies which patients are most likely to benefit from therapy. This work exemplifies R3, leveraging complex multimodal datasets, and touches on R5, as it includes external validation cohorts that hint at real-world applicability. The model’s stratification of patients also speaks to R6, suggesting a scalable framework that could be incorporated into clinical decision-making across institutions.

In type 2 diabetes, Shamanna et al. [52] used digital twins to predict postprandial glycemic responses and deliver personalized dietary recommendations. By tailoring nutrition based on continuous glucose monitoring and patient-specific data, this approach expands DT applications in line with R1 and demonstrates how integrated technologies can create adaptive, patient-centered interventions, satisfying R3. When the platform was tested in real-world settings, it provided early evidence of sustained benefits, supporting R5.

Joshi et al. [53] extended this personalized approach over a full year, combining nutrition, activity, and sleep recommendations. The study showed significant improvements not only in glycemic control but also in liver health and metabolic markers. This long-term perspective reinforces R1 and R5, illustrating how digital twins can maintain benefits over extended periods. It also exemplifies R3, by orchestrating multiple data types—nutrition, activity, and sleep—into a coherent, personalized treatment plan.

Finally, in oncology, Susilo et al. [54] developed systems-based digital twins to simulate dose–response relationships for a bispecific antibody in non-Hodgkin’s lymphoma. By creating virtual patient populations that account for heterogeneity, the study strengthens R2, offering reproducible methods, and supports R3 and R6, as the virtual populations enable scalable predictions for individualized therapy. The insights into tumor behavior and immune responses showcase the potential of DTS to guide adaptive dosing strategies in early-phase clinical trials.

Together, these studies illustrate how digital twins are progressively shaping healthcare. They broaden disease coverage (R1), provide reproducible frameworks (R2), integrate diverse and emerging technologies (R3), and, through real-world validation and extended follow-up, begin to demonstrate the longitudinal impact and scalability of DT interventions (R5–R6). Each trial, in its own way, illuminates the pathway from innovation to tangible clinical application. Table 3 summarizes the findings.

#### 4.3.2. Contributions from Primary, Conceptual, and Synthesized Evidence to Digital Twin Recommendations

This section extends the recommendation-guided synthesis (R1–R6) by analyzing how different categories of evidence—including primary studies, conceptual contributions, and non-structured secondary literature—differentially contribute to the development of digital twin (DT) applications in healthcare. Rather than treating these studies as a homogeneous body of evidence, the analysis explicitly accounts for differences in methodological rigor, abstraction level, and translational maturity.

Each contribution is therefore interpreted in relation to the six transversal recommendation domains (R1–R6), enabling a structured comparison of how heterogeneous evidence streams collectively support clinical translation, technological integration, methodological standardization, governance development, real-world validation, and interoperability. This approach allows emerging findings to be situated within a unified analytical framework, while preserving the epistemic distinctions between study types.

Table 4 summarizes the key contributions of these studies [55,56,57,58,59,60,61,62,63,64,65,66,67,68,69,70,71,72,73,74,75,76,77,78,79,80,81,82,83,84,85,86,87,88,89,90,91,92,93,94], highlighting the corresponding recommendation(s) they address.

Zhang et al. (2026) [55] introduce a new brain–digital twin dialogue paradigm using BCI, opening opportunities for personalized interventions in neuroscience and highlighting the need to extend DT applications to novel clinical areas (R1).

In diabetes care, Siddiqui & Khan (2026) [56] demonstrate that digital twin frameworks can enable predictive, personalized, and precision-guided management, reinforcing the need for longitudinal and real-world studies across diverse patient populations (R1, R5). Similarly, Rezaeitaleshmahalleh et al. (2026) [57] describe DT-supported adaptive endovascular devices in precision vascular medicine, emphasizing the integration of emerging technologies for real-time personalized interventions (R3).

Olawade et al. (2026a,b) [58,60] explore DT implementation in organ transplantation and oncology, respectively, showing potential for treatment personalization while underlining the importance of model standardization and data sharing to ensure reproducibility and scalability (R2, R6). Siddiqui & Khan (2026) [59] present a digital twin for *Mycobacterium tuberculosis* infection, integrating immune dynamics and pathogen adaptation, demonstrating how DTS can support precision therapy for infectious diseases (R1, R3).

On the oncology front, Carpino et al. (2026) [61] refine histologic subtypes and identify biomarkers linked to poor prognosis in cholangiocarcinoma through DT-based registries, advancing both precision treatment and population-level insights (R1, R5). Görtz (2026) [62] provides a comprehensive review of DTS, past, present, and future, emphasizing the need for ethical frameworks, governance, and interoperability across platforms (R4, R6). Yamamoto et al. (2026) [63] use biatrial DTS to study arrhythmogenic substrates in atrial fibrillation and ablation lesions, supporting precision interventions (R3, R5), while Kashani et al. (2026) [64] establish an international Delphi consensus on acute kidney injury to underpin AI-driven DTS in critical care nephrology, highlighting standardization, governance, and predictive modeling (R2, R4, R3).

Segal (2026) [65] reviews applications of DTS in asthma research, illustrating the promise of DTS to model disease mechanisms in populations with variable phenotypes, thereby supporting broader disease coverage (R1).

In drug development, Niarakis & Moingeon (2026) [66] demonstrate how DTS can accelerate target identification and drug discovery for immune-mediated disorders, emphasizing the integration of emerging technologies to improve precision therapy (R3). Forensic medicine is another emerging area: Joo & Vijayasimha (2026) [67] describe AI-assisted digital twins for injury documentation, highlighting translational applications of DTS beyond classical clinical settings (R1, R3).

Acute kidney injury (AKI) and neonatal sepsis illustrate the role of DTS in predictive, outcome-driven care. Cai et al. (2026) [68] introduce AKI-twinX, an explainable organ-structured DT for forecasting sepsis-related AKI trajectories, reinforcing the need for longitudinal and real-world evaluation (R5, R3). Similarly, Prunella et al. (2026) [69] present an evolutionary DT framework for optimal aminoglycoside dosing in neonates, showing how DTS can enable individualized treatment strategies (R1, R3, R5).

Mental health applications remain nascent: Verhees et al. (2026) [70] identify both milestones and hurdles for DTS in psychiatry, emphasizing ethical frameworks, standardization, and interoperability as critical for adoption (R2, R4, R6). Neuroscience DTS are further advanced by Di Antonio et al. (2026) [71], who use reservoir computing to linearize and forecast brain dynamics, illustrating methodological innovation and the integration of AI techniques (R3, R2).

Emergency care and reproductive health are also benefiting from DTS. Olawade et al. (2026) [72] discuss the role of DTS in modern emergency care, emphasizing real-time decision support and scalable clinical deployment (R5, R6), while Gorelova et al. (2026) [73] focus on ML-driven DTS for hormone biosensing in personalized infertility care, demonstrating integration of wearable and sensing technologies (R3, R1). Finally, Dinu et al. (2026) [74] explore operational bounds for hemodialysis modeling, highlighting data-driven approaches that enhance reproducibility and standardization (R2, R3).

Herrero et al. (2026) [75] demonstrate that DT-based glucose predictions can significantly improve glycemic control, emphasizing both patient-specific modeling and the need for longitudinal evaluation in diabetes care (R1, R5). Complementing this, Kiran et al. (2026) [80] use retrospective lifestyle data to predict type 2 diabetes onset via DTS, highlighting population-level personalization and predictive modeling (R1, R3).

In drug development and pharmacology, Ecker et al. (2026) [76] and Venkatapurapu et al. (2026) [84] show that DTS can predict drug side effects and accelerate discovery pipelines, stressing the importance of explainability, methodological standardization, and integration with AI technologies (R2, R3).

Cardiovascular applications are illustrated by Tanade et al. (2026) [77], who employ DTS for real-time peripheral revascularization planning in chronic limb-threatening ischemia, demonstrating precision-guided interventions and real-time clinical decision support (R3, R5). Similarly, Thangaraj et al. (2026) [78] propose a DT strategy to examine the implications of randomized clinical trials for real-world populations, bridging the gap between in-silico simulations and pragmatic outcomes (R5, R1).

Personalized medicine and federated learning approaches are further explored by Zheng et al. (2026) [79], who develop a patient-centric DT framework integrating hybrid knowledge distillation for class-incremental learning, advancing precision medicine applications (R1, R3). Infectious disease applications are also represented: Siddiqui & Khan (2026) [82] construct a DT for neonatal meningitis caused by *Escherichia coli* K1, highlighting opportunities for tailored interventions in high-risk populations (R1, R3).

Forensic medicine continues to expand DT applications, with Thali et al. (2026) [81] demonstrating AI-assisted injury detection using 3D avatars and interactive DT visualization, supporting translational and legal applications (R1, R3). Finally, Blum & Dy (2026) [83] propose a conceptual DT framework for nerve injury care, emphasizing individualized rehabilitation and long-term outcome tracking (R1, R5).

Alotaibi et al. (2026) [85] propose a cost-optimized DT framework that secures and streamlines patient data management, highlighting the importance of interoperability, scalability, and ethical governance for healthcare DTS (R4, R6).

Rehabilitation and chronic disease management are well represented. García-Rudolph et al. (2025) [86] review DT applications in stroke rehabilitation, detailing objectives, data sources, mechanisms, and outcomes, thus contributing to broader disease coverage and standardization of approaches (R1, R2). Similarly, Vallée (2025) [87] discusses DTS for cardiovascular disease, emphasizing sustainable, personalized care, reflecting the need for longitudinal and outcome-driven applications (R1, R5).

Pediatric and infectious disease applications further demonstrate the versatility of DTS. Esposito et al. (2025) [88] focus on pediatric infectious diseases, showing how virtual DTS enable personalized management strategies (R1, R3). Diabetes care continues to benefit from DT-based personalization, as highlighted by Cáceres-Gutiérrez et al. (2025) [89], who map architectures and outcomes, and Silva & Vale (2025) [90], who discuss bridging innovative DT concepts with clinical reality (R1, R3, R5).

Cancer and precision medicine are also strengthened by Moradi Kashkooli et al. (2025) [91], who integrate multiphysics modeling, imaging, and AI for personalized nanomedicine, laying foundations for clinical DT implementation (R3, R2, R5). Clinical trial innovation is exemplified by Akbarialiabad et al. (2025) [92], who show how DTS can enhance randomized trials, addressing reproducibility, real-world translation, and methodological rigor (R2, R5).

Finally, Park et al. (2025) [93] and Gu et al. (2025) [94] explore human DTS in pervasive healthcare and nursing, highlighting patient-centered monitoring, system integration, and cross-disciplinary applications, reinforcing both broader population coverage and interoperability needs (R1, R6).

### 4.4. International Contribution to Digital Twin Research in Healthcare

DT technologies are increasingly supported not only by academic research but also by international policy initiatives, strategic frameworks, and consensus efforts that help shape the future of healthcare innovation and governance. These contributions are crucial for establishing common definitions, data governance principles, interoperability standards, and pathways for clinical translation, enabling DTS to move from isolated research projects to scalable tools across health systems.

Assessing the international policy and governance landscape for DT technologies is inherently complex. A fully comprehensive review of all global documents, regulations, and initiatives is beyond the scope of this work. Nevertheless, selected international frameworks, strategic programs, and consensus efforts provide valuable insights into how digital twins are being conceptualized, regulated, and advanced as tools for personalized and predictive healthcare. This section therefore presents illustrative examples of how the international community is approaching DT development, validation, and clinical adoption, without attempting to provide an exhaustive inventory.

At the regulatory level, the U.S. Food and Drug Administration (FDA) has recognized the importance of digital twins within its Digital Health and Artificial Intelligence Glossary. A digital twin is defined as an information construct that mimics the structure, context, and behavior of a physical asset and can be dynamically updated through its lifecycle to inform decisions, including patient-specific clinical decision support, quality assessment, and in silico clinical trials [95]. This foundational definition helps align DT research with regulatory expectations for safety, decision support, and integration within digital health ecosystems.

In Europe, the European Virtual Human Twins (VHT) Initiative represents a major strategic effort to accelerate the development, validation, and adoption of DTS in health and care. Launched under the EU’s digital strategy, this initiative supports the creation of virtual human twin solutions that simulate physiological and pathological states across scales and clinical scenarios, enabling personalized prevention, tailored care pathways, and advanced clinical research platforms [96]. A related Statement of Intent on Development, Evidence, and Adoption in Healthcare Systems reflects a cross-sector commitment to build evidence, foster collaboration, and support integration of VHTs into national and regional health systems [97].

Moreover, broader EU data governance frameworks such as the European Health Data Space (EHDS) set the stage for secure, interoperable health data exchange across Member States. While not specific to digital twins, EHDS provides critical infrastructure for sharing and reusing health data that DT platforms can leverage for model building, validation, and cross-border research, addressing foundational challenges in data access, standardization, and governance.

Beyond policy initiatives, international multidisciplinary consensus statements are beginning to define how digital twins should be conceptualized, governed, and integrated into clinical practice. A recent globally relevant consensus published in npj Digital Medicine emphasizes the potential of DTS as pivotal innovations for personalized healthcare, and highlights the need for stakeholder engagement, evidence standards, ethical considerations, and infrastructure support to enable their broader adoption [98].

Taken together, these documents and initiatives contribute to an emerging global framework that supports the definition, validation, governance, and scaling of digital twins in healthcare. Importantly, they also clarify a key conceptual shift: DTS are not merely advanced data aggregation systems, but represent dynamic, model-driven infrastructures that require coordinated regulatory, technical, and ethical alignment to achieve clinical impact at scale. This alignment between policy, computation, and clinical practice is essential for translating DTS from experimental settings into robust, real-world healthcare applications across diverse health systems.

### 4.5. Rationale for the Narrative Review Design

The selection of a narrative review design was grounded in the intrinsic heterogeneity, interdisciplinarity, and rapid evolution of digital twin research in healthcare. The current literature spans a wide spectrum of evidence types, including randomized and controlled clinical trials, observational and real-world studies, in-silico simulations, artificial intelligence-driven predictive models, engineering frameworks, conceptual architectures, proof-of-concept implementations, and international policy and governance documents. In this context, the evidence base remains highly fragmented, methodologically non-uniform, and not yet sufficiently standardized to support exclusive reliance on rigid evidence synthesis methodologies without substantial loss of conceptual depth and translational insight.

A systematic review design was therefore considered inappropriate for the aims of this study, as it requires narrowly defined research questions, methodological homogeneity, and a degree of comparability that is not present in the current DT literature. Similarly, meta-analytic approaches were excluded due to the absence of sufficient outcome standardization and the predominance of heterogeneous study designs and reporting frameworks.

While systematic reviews are primarily designed to address narrowly defined research questions under conditions of methodological comparability and statistical aggregation, and scoping reviews are particularly suited for mapping the extent and nature of available literature, neither approach fully accommodates the need for interpretative integration across highly heterogeneous forms of evidence. In particular, scoping reviews—while useful for evidence mapping—remain primarily descriptive and do not enable the level of conceptual synthesis required to connect clinical, technological, and governance dimensions within a unified framework.

In contrast, the narrative review approach [99,100,101,102] allows for a more flexible, interpretative, and conceptually driven synthesis, which is particularly appropriate in emerging and rapidly evolving domains such as digital health and digital twin technologies. Narrative reviews have been widely recognized as a valid methodological approach when the aim is to integrate heterogeneous evidence, generate conceptual synthesis, and provide higher-order interpretation across diverse study designs rather than perform purely quantitative aggregation [99,100,102].

A central methodological justification for this choice lies in the necessity to integrate not only clinical evidence but also conceptual, technological, and policy-oriented knowledge within a unified analytical framework [101,102]. Digital twin research is characterized by continuous interaction between technological innovation and clinical translation, where methodological consolidation often follows rather than precedes innovation. As a result, the available evidence cannot be adequately captured through purely homogeneous aggregation strategies without overlooking critical dimensions such as governance, interoperability, ethical considerations, and system-level implementation challenges.

To address this complexity, the present study adopted a sequential two-step approach. In a first phase, recommendations were systematically extracted primarily from secondary literature, mainly systematic reviews and meta-analyses. These recommendations were then structured into transversal domains (R1–R6), representing key dimensions of digital twin development in healthcare, including clinical expansion, methodological standardization, technological integration, ethical governance, real-world validation, and interoperability.

In a second phase, these recommendations were used as an interpretative framework to guide a comparative analysis of primary evidence, including randomized controlled trials, clinical studies, observational investigations, real-world implementations, non-structured secondary studies, and international policy documents. This allowed for a structured cross-level synthesis in which empirical findings were not merely described but critically interpreted in relation to broader methodological and conceptual directions emerging from the literature. In particular, this approach enabled a differential analytical structure based on a sequential logic: analysis of structured secondary studies (primarily systematic reviews and meta-analyses), extraction of recommendations, complementary analysis of primary studies including RCTs and controlled clinical trials, and integration of non-structured secondary evidence and policy documents at national and international levels to examine the systemic dynamics activated through the identified recommendations.

This bidirectional analytical process created an iterative loop between evidence generation and conceptual synthesis. On one hand, secondary evidence informed the interpretation of primary studies by providing a structured set of analytical lenses. On the other hand, insights derived from primary research, non-structured secondary studies, and policy documents contributed to refining and contextualizing higher-order recommendations, ensuring that the synthesis remained grounded in empirical reality. Such an approach supports a more dynamic, context-sensitive, and system-aware understanding of digital twin development compared to static aggregation models.

Furthermore, the narrative design enabled the inclusion of international regulatory and governance perspectives, which are increasingly central to the development and deployment of digital twin systems in healthcare. These include emerging definitions, ethical frameworks, data governance strategies, interoperability initiatives, and health data infrastructures at both regional and global levels. Integrating these dimensions alongside clinical and technological evidence was essential to reflect the full ecosystem within which digital twin technologies are being developed and implemented.

Overall, the adoption of a narrative review design was not a methodological limitation but a deliberate and theory-driven choice aligned with the current maturity stage of the field. It enabled the construction of a comprehensive, multi-layered, and translational synthesis that connects clinical applications, computational methodologies, and policy frameworks within a unified interpretative structure. This integrative perspective provides a more complete understanding of digital twin systems in healthcare and supports the generation of actionable insights across multiple levels of healthcare innovation.

### 4.6. Shaping the Future of Healthcare with Digital Twins

DT technologies in healthcare are at an inflection point, moving from isolated research initiatives toward broader clinical and system-level applications. Despite the rapid growth of literature, primary studies, and international policy efforts, several challenges remain that shape the future research and implementation agenda. While it is impossible to predict every development, key trends and opportunities can be highlighted to guide the next phase of DT adoption.

First, expanding disease coverage and patient diversity remains a central priority. Current studies focus predominantly on diabetes, cardiovascular disease, neuro-oncology, and reproductive medicine, leaving many chronic, infectious, and pediatric conditions underexplored [21,22,23,24,25,26,38]. Future DT research should target broader clinical domains, including mental health, emergency care, and rare diseases, to enhance population-level personalization and support equitable healthcare access.

Second, interoperability and standardization are essential for enabling scalable DT platforms. International frameworks, such as the FDA Digital Health Glossary and the EU Virtual Human Twins initiative, provide early guidance for data standards, governance, and validation [85,86,87]. Building on these frameworks, future efforts should prioritize open-access model repositories, shared ontologies, and reproducible computational pipelines that facilitate multi-center studies, cross-border collaboration, and integration with emerging technologies.

Third, integration of emerging technologies will accelerate real-time predictive and precision interventions. Combining AI, wearable devices, multi-omics, Internet of Things (IoT), and virtual environments offers the potential to create fully adaptive, patient-centered DTS capable of simulating interventions, monitoring outcomes, and optimizing treatment pathways dynamically [35,36]. Advancing such integration will require careful attention to ethical and governance frameworks to protect privacy, ensure data ownership, and maintain patient trust [25,32].

Fourth, longitudinal, real-world studies are needed to validate clinical impact. While in-silico and short-term trials demonstrate promising predictive capabilities, few studies link DT outputs to actual patient outcomes over extended periods [21,23,34]. Large-scale, prospective trials and observational deployments will be critical to confirm clinical utility, cost-effectiveness, and operational feasibility across diverse healthcare settings.

Finally, policy alignment and global coordination will shape DT adoption. International contributions—from the FDA, EU initiatives, and consensus statements—highlight the importance of harmonizing regulatory approaches, standardizing validation criteria, and enabling cross-border research [95,96,97]. Future DT development should actively engage policymakers, clinicians, patients, and technology developers to ensure that innovations are safe, equitable, and scalable within real-world health systems.

In summary, the next decade of healthcare DT research will likely be defined by multidimensional expansion: across diseases, populations, technologies, and health systems. By addressing these interconnected priorities, the field can move from experimental implementations to clinically validated, ethically governed, and internationally interoperable digital twins that meaningfully improve patient care and system-level outcomes.

### 4.7. Limitations

This narrative review was conducted through a targeted search in PubMed, Scops, and Web of Science, focusing on systematic reviews and meta-analyses addressing patient-centered computational modeling and digital twin applications in the health domain. While relying on 3 single databases publications may have excluded some relevant studies, this strategy ensured a focus on high-quality, clinically relevant evidence. To complement these sources and provide a more comprehensive understanding, additional insights from primary studies, randomized controlled trials, non-systematic reviews, and policy documents were considered in the discussion. This integration allowed the identification of emerging trends, practical gaps, and opportunities that may not be fully captured by systematic reviews alone. By combining rigorous evidence with broader literature, the approach transforms inherent methodological limitations into a strength, providing a robust and clinically oriented synthesis.

## 5. Conclusions

From this comprehensive analysis, several key insights can be drawn. Digital twins and patient-specific computational models are increasingly applied in healthcare, supporting predictive, personalized, and adaptive interventions. Integration with AI enhances their capability to simulate clinical scenarios and optimize treatment strategies. While systematic reviews summarize validated evidence and well-established applications, the complementary examination of primary studies and non-systematic reviews shows that these studies are actively contributing to the stabilization of clinical evidence, supporting the consolidation of knowledge and the development of more robust patient-specific strategies. Overall, this combined approach highlights both the current state of evidence and the ongoing efforts to refine, validate, and implement digital twin technologies in clinical practice, emphasizing that further research is needed to fully realize their potential.

## Figures and Tables

**Table 1:** Summary of included studies on digital twin applications in healthcare. The table details each study’s focus, methods and technologies used, key results, and the primary conceptual contribution or identified pattern, highlighting how digital twins are applied across diverse clinical domains.

Reference	DT Focus	Methods/Technology	Results	Emerging Theme
Saeedian et al., [21]	Self-care support in adults with diet-related chronic conditions (T2D)	Systematic review; continuous glucose monitoring, wearable devices, dietary logs, AI-driven DT platforms, sometimes combined with human coaching	DT interventions improved multi-behavioural self-care, reduced or eliminated diabetes medications, increased physical activity, improved sleep, maintained glycemic control; evidence limited to T2D	Personalized, multi-behavioural self-care; integration of wearable and AI technologies
Singh et al., [22]	Neuro-oncology: tumor growth, treatment response	Systematic review; patient-specific computational models, mechanistic/biophysical frameworks, AI/hybrid models, MRI-based data	DTS simulated tumor behavior, radiation response, immune interactions, and drug transport; predictive accuracy high, but reproducibility, multimodal integration, external validation limited	Mechanistic + AI hybrid models; predictive modeling for therapy optimization
Vallée et al., [23]	Fertility, ART, and pregnancy	Systematic review; human and in-silico models, predictive/descriptive simulations	DTS applied to embryo selection, IVF modeling, placental physiology, pharmacokinetics, intrapartum monitoring; mostly theoretical, early-stage validation; risk of bias moderate-high	Early-stage clinical application; personalized reproductive simulations
Sarani Rad et al., [24]	Precision cardiology	Systematic review; mechanistic and AI/ML models, imaging, ECG/electrical signals; visualization mostly static	DTS applied to therapy planning, risk prediction, monitoring; benefits include improved decision-making, limited evidence on patient outcomes; barriers: model assumptions, computation, data quality, external validation, workflow integration	Patient-specific physiological modeling; precision therapy planning; integration challenges
Afshar et al., [25]	Healthcare: general DT applications	Umbrella review of systematic reviews; real-time data integration, predictive modeling	DTS enhance personalized care, operational efficiency, chronic disease management, surgical planning; barriers: data privacy, validation, high costs; ethical challenges require attention	Broad healthcare applications; focus on operational optimization and ethical considerations
Cappon & Facchinetti, [26]	Type 1 Diabetes	Systematic review; modeling glucose-insulin metabolism, twinning procedures, validation, consideration of physical activity, data for twinning	Focused on simulating glucose response to interventions; DTS not yet comprehensive multi-scale replicas; limitations and future directions identified	Disease-specific modeling; potential for precision therapy; limitations in full-scale personalization
Tubbs A, Vazquez EA [27]	DTS to improve diversity in clinical trials	Systematic review of 90 studies; DTS with AI for dynamic simulations, predictive analytics, recruitment optimization; bias/fairness assessment	DTS support personalized trial design, improve demographic representation, optimize recruitment; barriers: regulatory fragmentation, biased datasets, ethical/privacy concerns; inclusive data practices proposed	Equity and inclusion in clinical research through AI-enhanced DTS
John A, Alhajj R, Rokne J [28]	AI-based DTS for prostate cancer care	Systematic review of studies (2020–2025) on AI-driven DTS using ML/DL, image processing, predictive modeling, clinical decision support	DTS enhance predictive accuracy, enable early diagnosis, support individualized treatment; gaps: real-time integration, explainability, clinical validation	Precision oncology and patient-specific predictive modeling
Wah JNK [29]	AI-assisted robotic surgery with emerging digital twin-supported surgical planning	Systematic review and meta-analysis of 25 studies; AI-enabled robotic systems, digital twin-assisted planning, intraoperative AI analytics, vision models, and surgical automation	AI-assisted robotic surgery improved precision, operative efficiency, recovery outcomes, and complication reduction; digital twin technologies supported surgical simulation and preoperative planning	Enhanced surgical precision and efficiency via AI-robotic DTS
Lu M, Saeys W, Maryam M, et al. [30]	3D/4D digital human modeling (DHM) integrated with XR for rehabilitation	Systematic review of 16 experimental studies; 3D/4D DHM captured via 3D cameras/Wii remotes, integrated with VR games/avatar therapy; COSMIN & EBRO for quality	DHM-XR improves functional, physical, psychological, and general health outcomes; effective in neglect, anorexia, bulimia, T2D; variability in study parameters	XR-based personalized rehabilitation and functional recovery
Seth I, Lim B, Lu PYJ, et al. [31]	DTS for preoperative planning and intraoperative guidance in plastic surgery	Systematic review of 9 studies; DTS applied to reconstructive/cosmetic surgery using imaging and computational modeling	DTS improve surgical precision, reduce complications, enhance patient satisfaction; challenges: high cost, technical complexity, limited postoperative monitoring	Personalized surgical planning and intraoperative guidance
Sibanda K, Ndayizigamiye P, Twinomurinzi H [32]	NFTs for patient data management, supply chain, and DT development	Systematic review and thematic analysis of 19 papers; coding and six-step thematic analysis	Identified use cases in patient-centric data control, provenance tracking, and digital twin frameworks; prototypes dominate; challenges: blockchain dependence, interoperability, costs; research gaps in dual ownership, data pricing, open standards	Decentralized, secure, patient-controlled health data and NFT-enabled DTS
Lazarev AV, Kalininskaya AA [33]	Digital twins as components of digital healthcare	Systematic review of national and international literature	Analysis of DTS in patient modeling and organ-level simulations; examples from Russia and abroad; conceptual discussion on DT strategy and development	DT adoption in healthcare system management and strategic planning
Chumnanvej S, Chumnanvej S, Tripathi S [34]	DTS in neurosurgery	Systematic review of 25 RCTs and observational studies	DT applications improve neurosurgical navigation, robotics, and image-guided surgery; most studies low risk of bias; fewer post-operative complications compared to conventional methods	Personalized neurosurgical care and improved patient safety via DTS
Sheng B, Wang Z, Qiao Y, et al. [35]	DT trends in healthcare	Structural Topic Modeling (STM) of 94 papers (2018–2022)	Identified eight topics covering disease treatment and health enhancement; emphasis on AI, IoT, real-time services; research influenced by policies, COVID-19, emerging technologies	Quantitative insight into DT adoption, technological trends, and predictive modeling in healthcare
Shumba AT, Montanaro T, Sergi I, et al. [36]	Wearable tech and edge AI for chronic heart failure management	Systematic review of wearable devices and AI at the far edge	Wearables + edge AI support real-time, privacy-preserving patient monitoring; integration into IoT-aware infrastructures; challenges: architecture evaluation, interoperability	Edge-enabled personalized care and chronic disease management with DT-like monitoring
Rodero C, Baptiste TMG, Barrows RK, et al. [37]	Cardiac in-silico clinical trials (ISCTs)	Systematic review of 36 ISCT publications (2012–2022);	ISCTs assess interventions with variable validation practices; gaps: demographic reporting, uncertainty quantification, model/data sharing; software often unreported	Digital cardiac modeling for preclinical testing and standardized ISCT reporting
Mwanza J, Telukdarie A, Igusa T [38]	Industry 4.0 in low- and middle-income healthcare	Systematic review of 72 studies	Industry 4.0 adoption fragmented; mobile health and telemedicine dominate; gaps in AR, additive manufacturing, simulation, DTS	Optimizing healthcare in low-resource settings; DTS as emerging tools for system-level planning
Greggio et al., [39]	MRI-based digital twinning applications	Systematic review (PRISMA); 5 databases; 51 included studies; qualitative + quantitative synthesis; MRI radiography workflows; AI-assisted imaging pipelines; cardiac and oncologic imaging DT models	DTS mainly used in cardiac imaging, oncology diagnosis and therapy planning; strong focus on diagnosis, monitoring, and treatment optimization (63%); secondary applications include hardware/protocol optimization (20%), QA, cost-efficiency, training/education, and sustainability; evidence highlights early-stage adoption, with limited validation, interoperability issues, and underdeveloped radiographer-centered applications	Domain-specific DTS in radiology; transition from experimental MRI applications to structured clinical imaging ecosystems
Valdespino-Saldaña et al., [40]	Pediatric diabetes management	PRISMA systematic review; PubMed/Web of Science/BIREME; ML algorithms; CGM; closed-loop insulin delivery; telemedicine; digital education systems; DT-based predictive models	AI/DT-based systems improved HbA1c reduction, time-in-range extension, early complication detection, personalized insulin dosing, and patient self-management autonomy; DT and neural models enhanced risk stratification and predictive accuracy; key limitations: unequal access, small sample sizes, lack of long-term validation, and limited real-world deployment	AI-driven DT-enabled predictive endocrinology; shift toward participatory and personalized chronic disease management
Miozza et al., [41]	Digital divide in pharmaceutical digital transformation	Systematic review of 70 studies + topic modeling (Latent Dirichlet Allocation); synthesis of 16 clusters into 5 macro-domains; value-chain analysis	Digital transformation affects drug discovery, organizational redesign, manufacturing optimization, supply chain resilience, and patient-centric models; adoption of DT/AI uneven due to regulatory constraints, infrastructural gaps, organizational inertia, and socio-economic disparities; large firms and advanced systems benefit most, while SMEs and LMICs lag behind	Structural inequality in DT adoption; systemic governance and equity challenges in pharmaceutical innovation ecosystems
Haykal et al., [42]	Cosmetogenomics and AI-driven dermatology	PRISMA systematic review (2012–2025); PubMed/Scopus/Embase; genomics + proteomics + AI integration; SNP-based stratification; imaging + DT integration	AI + multi-omics enable personalized skincare via identification of SNPs linked to inflammation, oxidative stress, collagen degradation; DTS used to integrate clinical, imaging, and lifestyle data for treatment optimization (topicals, lasers, injectables); limitations include heterogeneous endpoints, limited diversity (skin phototypes), and lack of meta-analysis	AI–omics–DT convergence enabling precision dermatology and aesthetic personalization
Espinoza-Vinces et al., [43]	AI in headache medicine	PRISMA systematic review (2000–2025); PubMed, Scopus, Web of Science, Cochrane, DOAJ; QUADAS-2, PROBAST, NOS, AXIS bias tools	AI improves migraine diagnosis, subtype classification, treatment response prediction, and neuroimaging interpretation; DTS support simulation of disease progression and treatment scenarios; emerging tools include wearable biomarkers and synthetic data; major limitations: small datasets, bias, poor external validation, privacy concerns, and interpretability issues	Clinical AI/DT integration in neurology with strong ethical and patient–physician interaction implications
Faiella et al., [44]	Digital twins in radiology	Systematic review; multimodal imaging (abdominal, cardiac, musculoskeletal, oncologic, dental); AI + computational modeling + imaging fusion; QUADAS-2	DTS applied in oncology, cardiology, hepatic surgery, brain tumor characterization, scoliosis, portal hypertension, and cardiothoracic imaging; key functions include predictive modeling, treatment optimization, dosimetry, and radiographer training; limitations: computational cost, standardization gaps, and insufficient clinical validation	Radiology-wide DT expansion toward precision imaging ecosystems
Ringeval et al., [45]	Healthcare digital twin systems (meta-review)	PRISMA-ScR meta-review of 25 systematic reviews; thematic synthesis across PubMed, Embase, CINAHL, PsycINFO, Web of Science	Identified 3 main DT applications: personalized medicine, operational efficiency, medical research; persistent barriers include ethical concerns, data fragmentation, interoperability issues, scalability limitations, and lack of mature validation frameworks	Healthcare system-level DT integration and implementation barriers in digital health ecosystems
Shen et al., [46]	Population-level precision health via DTS	Systematic review across 8 databases; JBI quality assessment; quantitative content analysis of 12 studies	DTS improved personalized therapy, risk prediction, and chronic disease management (cancers, diabetes, MS, heart failure, dental conditions); overall effectiveness reported at ~80% positive outcomes; quality generally acceptable but heterogeneity high	Evidence for population-scale clinical effectiveness of DT-enabled precision healthcare
Asad et al., [47]	Human-centric industrial digital twins	Systematic literature review + VOSviewer keyword mapping; CPS, IoT, AI, VR/AR integration; Industry 5.0 frameworks	DTS enhanced human–machine interaction, ergonomics optimization, task allocation, training, and AI model development; enabling technologies include motion sensors, biological sensors, simulation platforms, and immersive visualization systems	Human-in-the-loop DT systems in Industry 5.0 cyber-physical environments
Paul et al., [48]	Digital human modeling and ergonomics 4.0	Qualitative meta-analysis; CPS + DHM + Industry 4.0 frameworks; semantic synthesis	DHM evolves into DT as a core component of Ergonomics 4.0, enabling integration of human behavior modeling, cyber-physical systems, and industrial automation; supports human–robot collaboration and system optimization	Foundational conceptual framework linking DTS, human modeling, and cyber-physical systems

**Table 2:** Broad categorization of digital twin contributions in healthcare. The table groups included studies into six overarching dimensions.

Broad Dimension	Representative Applications	Key Insights/Contribution	Representative References
Continuous and Personalized Patient Representation	Chronic disease management (diabetes, HF), pediatric diabetes, wearable-based monitoring, behavioral self-care systems, population precision health	DTS enable continuous, longitudinal patient modeling integrating physiological, behavioral, metabolic, and lifestyle data. They support adaptive self-care, glycemic control improvement, medication adjustment, and early complication detection. Evidence spans adult, pediatric, and population-level applications	[21,26,36,40,46]
Predictive and Simulation-Based Healthcare	Oncology, neuro-oncology, prostate cancer, fertility, cardiology, in-silico trials, precision medicine modeling	DTS enable predictive modeling of disease progression and treatment response using AI, mechanistic and hybrid models. In-silico trials support virtual experimentation, improved inclusivity, and accelerated clinical research across oncology, cardiology, and reproductive medicine	[22,23,24,27,28,37,46]
Procedural and Interventional Support	Neurosurgery, plastic surgery, robotic-assisted surgery, radiology, MRI, interventional imaging, XR rehabilitation	DTS enhance procedural planning, intraoperative navigation, and post-operative evaluation. Imaging-based DTS improve diagnostic precision and treatment optimization. XR and 3D/4D models support rehabilitation and surgical simulation training	[29,30,31,34,39,44]
Integration within Digital Health Ecosystems	AI platforms, blockchain/NFT systems, pharmaceutical ecosystems, interoperable healthcare infrastructures	DTS are embedded in broader ecosystems integrating AI, blockchain, IoT, advanced analytics, and human-centric cyber-physical systems. These systems enable interoperability, secure data sharing, patient data governance, and integration across pharmaceutical, healthcare, and human-in-the-loop value chains	[25,32,33,35,41,45,47,48]
Implementation, Scalability, and System-Level Impact	Health systems integration, LMIC adoption, regulatory frameworks, digital divide, infrastructure readiness	Adoption depends on governance, regulatory alignment, infrastructure maturity, and equity of access. Digital divide and fragmentation limit scalability, especially in low-resource settings. System-level transformation requires interdisciplinary coordination	[25,33,38,41,45,46]
Cross-Domain Enabling Technologies and Methodological Foundations	AI + genomics, multi-omics, headache medicine AI, radiology AI, human-centric DTS, CPS, meta-analyses	DTS increasingly rely on hybrid AI-physics systems, multi-omics integration, and cyber-physical architectures. Applications extend to dermatology, headache medicine, radiology, and industrial ergonomics. Methodological studies highlight fragmentation but growing convergence of DT paradigms	[41,42,43,44,47,48]

**Table 3:** Contribution of Clinical Trials to Digital Twin Recommendations in Healthcare.

Study	Clinical Context	Key Findings/DT Contribution	Related Recommendation(s)
Builes-Montaño et al., 2025 [50]	Type 1 Diabetes	DT-enabled decision support for insulin dosing; improved time-in-range; fewer hypoglycemic events; reproducible framework	R1, R2, R3
Saad et al., 2025 [51]	Early-stage NSCLC (Oncology)	Causal AI model integrating radiomics & clinical data for patient selection in immunotherapy + SABR; external validation; scalable framework	R3, R5, R6
Shamanna et al., 2024 [52]	Type 2 Diabetes	DT predicts postprandial glycemia; personalized dietary recommendations; real-world deployment; adaptive patient-centered intervention	R1, R3, R5
Joshi et al., 2023 [53]	Type 2 Diabetes with MAFLD	1-year study combining nutrition, activity, sleep; improved glycemic control, liver & metabolic markers; long-term follow-up	R1, R3, R5
Susilo et al., 2023 [54]	Non-Hodgkin’s Lymphoma	Systems-based DTS for bispecific antibody dose–response; virtual patient populations; reproducible & scalable predictive models	R2, R3, R6

**Table 4:** Contribution of the selected studies to Digital Twin Recommendations in Healthcare.

Study	Key Contribution	Related Recommendation(s)
Zhang et al., 2026 [55]	Brain–DT dialogue via BCI; personalized neuroscience applications	R1
Siddiqui & Khan, 2026 [56]	Predictive, personalized diabetes management	R1, R5
Rezaeitaleshmahalleh et al., 2026 [57]	Adaptive endovascular devices; integration of emerging technologies	R3
Olawade et al., 2026a [58]	DT in organ transplantation; clinical translation pathways	R2, R6
Siddiqui & Khan, 2026 [59]	DT for TB infection; immune dynamics and pathogen adaptation	R1, R3
Olawade et al., 2026b [60]	DT in oncology; personalized treatment strategies	R2, R6
Carpino et al., 2026 [61]	DT-based registry for cholangiocarcinoma; biomarker identification	R1, R5
Görtz, 2026 [62]	DT evolution; governance and interoperability needs	R4, R6
Yamamoto et al., 2026 [63]	Biatrial DT for AF; precision interventions and risk prediction	R3, R5
Kashani et al., 2026 [64]	Delphi consensus for AI-driven DTS in critical care nephrology	R2, R3, R4
Segal, 2026 [65]	DTS in asthma research; modeling variable phenotypes	R1
Niarakis & Moingeon, 2026 [66]	DTS for drug target identification and development	R3
Joo & Vijayasimha, 2026 [67]	Forensic DTS for AI-assisted injury documentation	R1, R3
Cai et al., 2026 [68]	AKI-twinX; organ-structured DT for sepsis AKI forecasting	R3, R5
Prunella et al., 2026 [69]	Evolutionary DT for neonatal drug dosing	R1, R3, R5
Verhees et al., 2026 [70]	DTS in mental health; milestones and hurdles	R2, R4, R6
Di Antonio et al., 2026 [71]	Brain DTS using reservoir computing for forecasting	R2, R3
Olawade et al., 2026 [72]	DTS in emergency care; real-time decision support	R5, R6
Gorelova et al., 2026 [73]	ML-driven DT for hormone biosensing in infertility	R1, R3
Dinu et al., 2026 [74]	Data-driven hemodialysis DT modeling	R2, R3
Herrero et al., 2026 [75]	DT-based glucose prediction; improved glycemic control	R1, R5
Ecker et al., 2026 [76]	DTS for drug side effect prediction	R2, R3
Tanade et al., 2026 [77]	Real-time DT for peripheral revascularization planning	R3, R5
Thangaraj et al., 2026 [78]	DT bridging RCTs and real-world populations	R5, R1
Zheng et al., 2026 [79]	Patient-centric DT with hybrid learning	R1, R3
Kiran et al., 2026 [80]	DT for type 2 diabetes onset prediction	R1, R3
Thali et al., 2026 [81]	Forensic 3D avatar DTS; injury detection	R1, R3
Siddiqui & Khan, 2026 [82]	DT for neonatal *E. coli* K1 infection	R1, R3
Blum & Dy, 2026 [83]	Conceptual DT framework for nerve injury care	R1, R5
Venkatapurapu et al., 2026 [84]	DTS in drug discovery pipelines	R2, R3
Alotaibi et al., 2026 [85]	Secure cost-optimized DT data management	R4, R6
García-Rudolph et al., 2025 [86]	DTS in stroke rehabilitation	R1, R2
Vallée, 2025 [87]	Cardiovascular DTS for sustainable care	R1, R5
Esposito et al., 2025 [88]	Pediatric infectious disease DTS	R1, R3
Cáceres-Gutiérrez et al., 2025 [89]	DT frameworks for diabetes care	R1, R3, R5
Silva & Vale, 2025 [90]	Bridging DT innovation and clinical reality	R1, R3, R5
Moradi Kashkooli et al., 2025 [91]	Multiphysics AI DTS for cancer nanomedicine	R2, R3, R5
Akbarialiabad et al., 2025 [92]	DTS enhancing clinical trials	R2, R5
Park et al., 2025 [93]	Human DTS in pervasive healthcare	R1, R6
Gu et al., 2025 [94]	DTS in nursing; monitoring systems	R1, R6

## Data Availability

No new data were generated.

## Supplementary Materials

The following supporting information can be downloaded at: https://www.mdpi.com/article/10.3390/medsci14020330/s1. Table S1. Consensus report.
