# Supplementary material for: Narrative Review of Digital Twins in the Health Domain: Development, Application, and Evidence Consolidation
Source: Med Sci (Basel). 2026 Jun 18;14(2):330. doi: 10.3390/medsci14020330 (PMC13304318; doi:10.3390/medsci14020330)
Supplement: Supplementary file 1 [file medsci-14-00330-s001.zip › medsci-4265357-supplementary.pdf]

medical sciences

MDPI

Review

Narrative Review of Digital Twins in the Health Domain: Development, Application, and Evidence Consolidation

SUPPLEMENTARY MATERIAL

Daniele Giansanti [1,*] and Claudia Cosenza [2]

S1. Narrative Review Selection Flow (PRISMA-inspired)

Although this work is a narrative review and not a systematic review, a PRISMA-inspired approach was used to improve transparency in study identification and selection.

The literature search was conducted across three electronic databases (PubMed, Scopus, and Web of Science), using predefined search terms related to digital twins and associated concepts in healthcare. The initial search yielded 511 records (PubMed: 131; Scopus: 220; Web of Science: 160).

After removal of duplicates, 392 records were retained for screening. Titles and abstracts were reviewed for relevance to digital twin applications in healthcare, resulting in the exclusion of records not aligned with the scope of this narrative synthesis.

Full-text assessment was subsequently performed on 78 eligible studies, with exclusions based on lack of alignment with the focus of this narrative review or insufficient relevance to digital twin applications in healthcare.

Following full-text assessment, 28 peer-reviewed studies were included in the final synthesis. Study selection was conducted through discussion and consensus among the authors to ensure coherence with the objectives of the review. All inclusion decisions were documented in a selection log, an excerpt of which is provided in Table S1 of the Supplementary Materials.

This approach was adopted to ensure clarity and reproducibility of the selection pathway while preserving the interpretative nature of a narrative synthesis.

Table s1. Consensus report.

Article	Justification
Saeedian Y, Wright C, Jansons P, Shen Y, Zhang Y, Maddison R. Digital twin technologies for supporting self-care in adults with diet-related chronic conditions: a systematic review. Int J Med Inform. 2026 Mar 22;214:106404. doi:10.1016/j.ijmedinf.2026.106404. Epub ahead of print. PMID:41880918.	Recent systematic review focused on digital twin applications for self-care and chronic disease management; relevant to patient-centered digital health and behavioral support systems.
Singh A, Qureshy FA, Kurtz A, Bhattacharya M, Prasanna P, Singh G. Digital Twins in Neuro-Oncology: A Systematic Review of Current Implementations, Technical Strategies, and Clinical Applications. Radiol Imaging Cancer. 2026 Mar;8(2):e250567. doi:10.1148/rycan.250567. PMID:41823607.	Systematic review on digital twin implementations in neuro-oncology, covering clinical applications and computational strategies in precision oncology.

Vallée A, Moawad G, Feki A, Ayoubi JM. Digital twins in fertility, assisted reproductive technology and pregnancy: a systematic review. Reprod Biomed Online. 2026 Mar;52(3):105281. doi:10.1016/j.rbmo.2025.105281. Epub 2025 Sep 29. PMID:41678856.	Systematic review of digital twin applications in reproductive medicine and pregnancy monitoring, highlighting precision medicine approaches in maternal–fetal health.
Sarani Rad F, Bitaraf E, Jafarpour M, Li J. Technologies, Clinical Applications, and Implementation Barriers of Digital Twins in Precision Cardiology: Systematic Review. JMIR Cardio. 2026 Jan 8;10:e78499. doi:10.2196/78499. PMID:41505790; PMCID:PMC12782626.	Systematic review addressing digital twin technologies in precision cardiology, including clinical use cases and implementation barriers in cardiovascular care.
Afshar M, Moradkhani A, Soheili M, Tavakkol M, Moradi Y, Kohan HG. Digital Twins and Health Care: an Umbrella Review. J Med Syst. 2025 Dec 20;49(1):186. doi:10.1007/s10916-025-02322-9. PMID:41420670.	Umbrella review synthesizing evidence on digital twins in healthcare, providing a high-level overview of clinical applications and system-level integration.
Cappon G, Facchinetti A. Digital Twins in Type 1 Diabetes: A Systematic Review. J Diabetes Sci Technol. 2025 Nov;19(6):1641-1649. doi:10.1177/19322968241262112. Epub 2024 Jun 17. PMID:38887022; PMCID:PMC11572256.	Systematic review on digital twin applications in type 1 diabetes, focusing on glucose modeling, decision support, and personalized disease management.
Tubbs A, Vazquez EA. Digital twins in increasing diversity in clinical trials: A systematic review. J Biomed Inform. 2025 Sep;169:104879. doi:10.1016/j.jbi.2025.104879. Epub 2025 Aug 8. PMID:40784603.	Systematic review exploring digital twin use in improving diversity and representativeness in clinical trials, supporting methodological innovation in clinical research design.
John A, Alhajj R, Rokne J. A systematic review of AI as a digital twin for prostate cancer care. Comput Methods Programs Biomed. 2025 Aug;268:108804. doi:10.1016/j.cmpb.2025.108804. Epub 2025 May 6. PMID:40347618.	Systematic review on AI-driven digital twins in prostate cancer care, emphasizing predictive modeling and clinical decision support systems.
Wah JNK. The rise of robotics and AI-assisted surgery in modern healthcare. J Robot Surg. 2025 Jun 20;19(1):311. doi:10.1007/s11701-025-02485-0. PMID:40540146; PMCID:PMC12181090.	Review on AI-assisted and robotic surgery systems, relevant to digital twin-enabled surgical planning, training, and clinical automation workflows.
Lu M, Saeys W, Maryam M, Gjeleshi I, Nazarahari H, Truijen S, Scataglini S. Using 3D and 4D digital human modeling in extended reality-based rehabilitation: a systematic review. Front Bioeng Biotechnol. 2025 Mar 12;13:1496168. doi:10.3389/fbioe.2025.1496168. PMID:40144388; PMCID:PMC11937100.	Systematic review on 3D/4D digital human modeling in XR-based rehabilitation, highlighting strong educational and training implications in clinical rehabilitation and digital health learning environments.
Seth I, Lim B, Lu PYJ, Xie Y, Cuomo R, Ng SK, Rozen WM, Sofiadellis F. Digital Twins Use in Plastic Surgery: A Systematic Review. J Clin Med. 2024 Dec 23;13(24):7861. doi:10.3390/jcm13247861. PMID:39768784; PMCID:PMC11728120.	Systematic review on digital twin applications in plastic surgery, focusing on surgical planning, personalization, and clinical workflow optimization.
Sibanda K, Ndayizigamiye P, Twinomurinzi H. Non-fungible tokens (NFTs) in healthcare: a thematic analysis and research agenda. Front Digit Health. 2024 Jun 11;6:1377531. doi:10.3389/fdgth.2024.1377531. PMID:38919876; PMCID:PMC11196843.	Thematic analysis on NFTs in healthcare, relevant to emerging digital health infrastructures, data ownership, and decentralized health innovation ecosystems.
Lazarev AV, Kalininskaya AA. [The digital health care and digital twins as its components: the systematic review]. Probl Sotsialnoi Gig Zdravookhranenniiai Istor Med. 2024 May;32(3):318-324. doi:10.32687/0869-866X-2024-32-3-318-324. PMID:39003534.	Systematic review discussing digital healthcare and digital twins as core components of modern health system transformation and digital infrastructure development.
Chumnanvej S, Chumnanvej S, Tripathi S. Assessing the benefits of digital twins in neurosurgery: a systematic review. Neurosurg Rev. 2024 Jan 18;47(1):52. doi:10.1007/s10143-023-02260-5. PMID:38236336.	Systematic review on digital twin applications in neurosurgery, emphasizing surgical planning, risk reduction, and precision neurosurgical interventions.

Sheng B, Wang Z, Qiao Y, Xie SQ, Tao J, Duan C. Detecting latent topics and trends of digital twins in healthcare: A structural topic model-based systematic review. Digit Health. 2023 Oct 12;9:20552076231203672. doi:10.1177/20552076231203672. PMID:37846404; PMCID:PMC10576938.	Topic modeling-based systematic review identifying research trends in healthcare digital twins, providing macro-level insight into field evolution and emerging applications.
Shumba AT, Montanaro T, Sergi I, Bramanti A, Ciccarelli M, Rispoli A, Carrizzo A, De Vittorio M, Patrono L. Wearable Technologies and AI at the Far Edge for Chronic Heart Failure Prevention and Management: A Systematic Review and Prospects. Sensors (Basel). 2023 Aug 3;23(15):6896. doi:10.3390/s23156896. PMID:37571678; PMCID:PMC10422393.	Systematic review on wearable technologies and AI for heart failure management, relevant to digital twin-enabled remote monitoring and patient-centered digital health systems.
Rodero C, Baptiste TMG, Barrows RK, Keramati H, Sillett CP, Strocchi M, Lamata P, Niederer SA. A systematic review of cardiac in-silico clinical trials. Prog Biomed Eng (Bristol). 2023 Jul 1;5(3):032004. doi:10.1088/2516-1091/acdc71. PMID:37360227; PMCID:PMC10286106.	Systematic review of in-silico cardiac clinical trials, representing a key methodological foundation for digital twin development in cardiovascular modeling and simulation-based medicine.
Mwanza J, Telukdarie A, Igusa T. Impact of industry 4.0 on healthcare systems of low- and middle-income countries: a systematic review. Health Technol (Berl). 2023;13(1):35-52. doi:10.1007/s12553-022-00714-2. PMID:36644409; PMCID:PMC9822693.	Systematic review on Industry 4.0 impacts in healthcare systems, highlighting digital transformation, infrastructure constraints, and adoption challenges in LMIC contexts.
Saeedian Y, Wright C, Jansons P, Shen Y, Zhang Y, Maddison R. Digital twin technologies for supporting self-care in adults with diet-related chronic conditions: a systematic review. Int J Med Inform. 2026 Mar 22;214:106404. doi:10.1016/j.ijmedinf.2026.106404. Epub ahead of print. PMID:41880918.	Recent systematic review (2026) directly relevant to digital twin applications in healthcare; addresses self-care in chronic conditions.
Greggio J, Stogiannos N, Stewart KL, Srivastava D, Hirani SP, Hilton S, Weldon SM, Malamateniou C. Exploring digital twinning in MRI: A systematic review of current applications, barriers, and future opportunities. Radiography (Lond). 2026 Apr 16;32(4):103413. doi:10.1016/j.radi.2026.103413. Epub ahead of print. PMID:41996784.	Recent systematic review focused on digital twinning in MRI, outlining applications, barriers, and future clinical opportunities in medical imaging.
Valdespino-Saldaña E, Altamirano-Bustamante NF, Calzada-León R, Revilla-Monsalve C, Altamirano-Bustamante MM. Artificial Intelligence-Driven Transformation of Pediatric Diabetes Care: A Systematic Review and Epistemic Meta-Analysis of Diagnostic, Therapeutic, and Self-Management Applications. Int J Mol Sci. 2026 Jan 13;27(2):802. doi:10.3390/ijms27020802. PMID:41596449; PMCID:PMC12841495.	Systematic review and meta-analysis on AI-driven transformation in pediatric diabetes care, covering diagnostic, therapeutic, and self-management applications.
Miozza M, Lombardo SM, Galvagno M, Magni L. Bridging the digital divide in the pharmaceutical industry: A future research agenda. Soc Sci Med. 2026 Jan;389:118832. doi:10.1016/j.socscimed.2025.118832. Epub 2025 Nov 27. PMID:41338043.	Relevant to digital transformation in the pharmaceutical sector, addressing structural barriers and future research directions.
Haykal D, Flament F, Amar D, Cartier H, Kourosh AS, Lee DH, Rowland-Payne C. Cosmetogenomics unveiled: a systematic review of AI, genomics, and the future of personalized skincare. Front Artif Intell. 2025 Nov 10;8:1660356. doi:10.3389/frai.2025.1660356. PMID:41293572; PMCID:PMC12640980.	Systematic review exploring AI and genomics integration for personalized skincare and emerging digital health applications.
Espinoza-Vinces C, Martínez MC, Atorrasagasti-Villar A, Rodríguez MDMG, Ezpeleta D, Irimia P. Artificial intelligence in headache medicine: between automation and the doctor-patient relationship. A systematic review. J Headache Pain. 2025 Sep 2;26(1):192. doi:10.1186/s10194-025-02143-8. PMID:40898015; PMCID:PMC12406602.	Systematic review on AI applications in headache medicine, focusing on clinical automation and implications for patient care.
Faiella E, Pileri M, Ragone R, Grasso RF, Zobel BB, Santucci D. Digital twins in radiology: A systematic review of applications, challenges, and future	Systematic review addressing applications and challenges of digital

perspectives. Eur J Radiol. 2025 Aug;189:112166. doi:10.1016/j.ejrad.2025.112166. Epub 2025 May 9. PMID:40382804.	twins in radiology and future clinical perspectives.
Ringeval M, Etindele Sosso FA, Cousineau M, Paré G. Advancing Health Care With Digital Twins: Meta-Review of Applications and Implementation Challenges. J Med Internet Res. 2025 Feb 19;27:e69544. doi:10.2196/69544. PMID:39969978; PMCID:PMC11888003.	Meta-review summarizing digital twin applications in healthcare and discussing implementation challenges and adoption barriers.
Shen MD, Chen SB, Ding XD. The effectiveness of digital twins in promoting precision health across the entire population: a systematic review. NPJ Digit Med. 2024 Jun 3;7(1):145. doi:10.1038/s41746-024-01146-0. PMID:38831093; PMCID:PMC11148028.	Systematic review evaluating the effectiveness of digital twins in precision health and population-level applications.
Asad U, Khan M, Khalid A, Lughmani WA. Human-Centric Digital Twins in Industry: A Comprehensive Review of Enabling Technologies and Implementation Strategies. Sensors (Basel). 2023 Apr 12;23(8):3938. doi:10.3390/s23083938. PMID:37112279; PMCID:PMC10146632.	Comprehensive review of human-centric digital twin technologies, including industrial and cyber-physical implementations, focusing on enabling technologies, human modeling frameworks, and implementation strategies with potential relevance to healthcare-oriented digital twin applications..
Paul G, Abele ND, Kluth K. A Review and Qualitative Meta-Analysis of Digital Human Modeling and Cyber-Physical-Systems in Ergonomics 4.0. IISE Trans Occup Ergon Hum Factors. 2021 Jul-Dec;9(3-4):111-123. Epub 2021 Aug 30. PMID:34380380.	Review and meta-analysis on digital human modeling and cyber-physical systems in Ergonomics 4.0, relevant to human-centered digital twin evolution.

https://doi.org/10.3390/xxxxx
